# Capturing naturalistic thoughts using a precision experience sampling idiographic approach

**DOI:** 10.1186/s41235-026-00728-8

**Published:** 2026-04-15

**Authors:** Julia W. Y. Kam, Sairamya Nanjappan Jothiraj, Emily Beauchemin, Nabil Al Nahin Ch, Laura K. Allen, Jolie B. Wormwood, Caitlin Mills

**Affiliations:** 1 Department of Psychology, University of Calgary, Calgary, Canada; 2 Hotchkiss Brain Institute, University of Calgary, Calgary, AB Canada; 3 Department of Educational Psychology, University of Minnesota, Minneapolis, USA; 4 Department of Psychology, University of New Hampshire, Durham, USA

**Keywords:** Precision experience sampling, Spontaneous thoughts, Mind wandering, Idiographic, Nomothetic, Machine learning

## Abstract

The existing literature on naturalistic thoughts has offered insights into the general patterns of thoughts common across large groups of participants. However, little is known about individual variability in thoughts. One approach to understanding variation within individuals is precision experience sampling, an idiographic approach that involves sampling inner experiences across multiple sessions and/or timepoints. This creates a comprehensive portrayal of an individual’s thoughts across time and context, which in turn facilitates person-specific predictions of their thoughts. The current study therefore used precision experience sampling to examine individual variations in naturalistic thoughts as a function of ongoing task. We implemented 7 sessions per participant (n = 7, idiographic group), resulting in 49 datasets. We verified that the descriptives of thoughts and task-modulatory effects of thoughts in this group were comparable to a larger cohort of participants (n = 49, nomothetic group) who each completed one session. Both groups were asked to complete whatever task they wished on the laboratory computer and to occasionally report their current task and numerous thought dimensions. Our results revealed considerable individual differences in the modulatory effects of task on thought dimensions, such that individuals engaged in different types of thoughts under different task contexts, underscoring the importance of considering both individual and contextual factors. They also indicated that patterns observed at the group level did not always accurately represent individual level patterns. Furthermore, applying machine learning algorithms on reports of the task-at-hand reliably detected all thought dimensions, with superior classification performance in the idiographic compared to nomothetic group. Overall, our study demonstrates the idiosyncratic effects of task on naturalistic thoughts and highlights the value of precision experience sampling in improving person-specific predictions of thoughts, which has important methodological and clinical implications.

**Supplementary Information:**

The online version contains supplementary material available at 10.1186/s41235-026-00728-8.

## Significance statement

The human mind has a remarkable ability to engage in thoughts that allow us to reminiscence the past, make decisions about the future and contemplate important scientific concepts. Research has thus far offered insights into general patterns of thoughts that are common across large groups of individuals. However, understanding individual variations in our thoughts across time and context is equally important, especially for making person-specific predictions of thoughts. In examining whether there are variations in naturalistic thoughts within and across individuals across different task contexts, our results revealed considerable individual differences in the types of thoughts they have across different tasks, highlighting idiosyncratic relationships between what we do and what we think about. Furthermore, our results demonstrated that we can better predict the occurrence of various types of thoughts using machine learning in individuals using their own data compared to group-level data, underscoring the value of obtaining large amounts of data within individuals in order to improve person-specific predictions of thoughts. These results have important implications in clinical conditions, educational contexts and beyond. Not only are our thoughts closely tied to everyday cognitive processes including creative problem solving, certain types of thoughts are also closely linked to conditions such as ADHD and depression. Combined with the appropriate intervention, these person-specific predictions of thoughts serve as an important step toward increasing thoughts that are beneficial (e.g., creative thoughts) or decreasing thoughts that are harmful (e.g., ruminative thoughts).

**Supplementary Information:**

The online version contains supplementary material available at 10.1186/s41235-026-00728-8.

## Introduction

As a key aspect of the human experience, our ongoing stream of thoughts unfolds over time and varies in content, dynamics and modality. Thus far, most empirical studies have advanced our understanding of the general patterns of thought by aggregating data to identify experiences that are common to large groups of participants. This *nomothetic* approach, however, is limited insofar as our ongoing experiences of thoughts are likely to be uniquely personal and specific to our own lived experiences, which is not necessarily reflected by the “average” experience. This necessitates an approach that is better suited for capturing such intra-individual specificity. To that end, the *idiographic* approach involves acquiring multiple datasets from each individual across different sessions or timepoints. In the context of thoughts, this entails implementing precision experience sampling to acquire numerous reports of individuals’ ongoing stream of thoughts across different sessions, and therefore across contexts, as well as mental and emotional states. In leveraging the multiple data points available for each individual, this approach allows us to assess the types of thoughts that are unique to each individual and facilitates person-specific predictions about their thoughts. This study therefore aims to examine individual variations in naturalistic thoughts using precision experience sampling as an idiographic approach.

### General patterns of thoughts

Our stream of thought occupies most, if not all, of our waking moments and has widespread consequences for our daily life, with important clinical implications. There are numerous dimensions upon which thoughts can vary, with evidence converging on several dimensions that commonly occur and are robustly linked to functional outcomes such as affective well-being. To date, much of the research efforts on thoughts have focused on the task-relatedness thought dimension, with evidence indicating that off-task thought occurs during approximately 35% of our awake hours in daily life (see (Kawashima et al., 2023) for a meta-analysis). Not only are they prevalent, but off-task thoughts have also been consistently shown to impair task performance (see (Randall et al., 2014; Wong et al., 2022) for meta-analyses), and they are linked to negative affect when they occur unintentionally (Seli et al., 2016; see (Kam et al., 2024) for a meta-analysis). Although task-unrelated thoughts are often assumed to be internally oriented, these two thought dimensions have been associated with different brain regions suggesting that they represent different dimensions of thoughts (Stawarczyk et al., 2011). Internally oriented thoughts refer to thoughts focused internally toward one’s thoughts, memories, emotions and other internal representations, at the exclusion of anything in our immediate external environment (Kam et al., 2019, 2021). These two thought dimensions have clinical implications, as demonstrated in a recent study that found individuals with social anxiety disorder are more likely to report internally oriented but not externally oriented off-task thoughts compared to controls (Arch et al., 2021).

Studies taking a closer look into the content of thoughts have also found that individuals commonly report thinking about themselves or others (Baird et al., 2011; Mckeown et al., 2021; Mulholland et al., 2023; Ruby et al., 2013). This dimension of thought has been differentially associated with affective well-being. For example, thoughts about the self (in the future) have been linked to positive affect (Ruby et al., 2013; Smallwood et al., 2021), whereas thoughts about others (in the past) were linked to negative affect (Ruby et al., 2013).

In addition to thought content, previous work has examined the dynamics of thoughts, as conceptualized in the Dynamic Framework of Thought (Christoff et al., 2016). On one end of the spectrum, thoughts can freely move from one topic to another. This type of thought occurs during about 30% to 50% of our awake hours in everyday life (Mills et al., 2018) and is associated with positive affect (Mills et al., 2021; Thiemann et al., 2023). Moreover, freely moving thought has been implicated in clinical conditions, such that individuals with attention deficit/hyperactivity disorder report higher levels of these thoughts that move from topic to topic (Mowlem et al., 2019; Raffaelli et al., 2025). On the other end of the spectrum, thoughts can also be constrained to a given topic. Specifically, thoughts can be constrained deliberately, in which they are goal-directed toward a topic, and thoughts can also be constrained on a topic such that we feel stuck on a topic and cannot stop thinking about it even if we tried. This latter category of sticky thought is conceptually similar to ruminative thoughts, which have been closely linked to clinical disorders such as anxiety and depression (Olatunji et al., 2013; Spasojević & Alloy, 2001).

Finally, our thoughts can also differ in form, such that they can occur in the visual modality such as imagining a beautiful beach or the auditory modality such as having an inner dialogue with oneself. Past literature has reported that the modality of our thoughts is modulated by the modality of the task-at-hand (Choi et al., 2017), and that thought modality is not stable over time (Öncel et al., 2024). Collectively, these studies highlight some of the most common thought dimensions, offering a multi-faceted portrayal of the nature of our ongoing thoughts and inform their functional consequences in our everyday life.

The vast majority of these past studies on thoughts have primarily offered insights into the general patterns of thoughts by examining aggregate data at the group level. This nomothetic approach focuses on identifying general patterns about the population and rests on an assumption of homogeneity across individuals (Molenaar, 2004; Molenaar & Campbell, 2009). Although it is informative to understand the thought patterns that are common across individuals, recent studies have identified individual differences in different dimensions of our thoughts (Welhaf et al., 2020; Zanesco et al., 2024), highlighting the importance of also understanding variations across, and within, individuals.

### Individualized patterns of thoughts

Thus far, only a handful of studies have examined ongoing thoughts at the individual level, revealing patterns of thoughts and their correlates that are unique to each individual. For instance, (Hurlburt & Akhter, 2006) aimed to develop highly accurate accounts of individuals’ inner experiences by sampling their experience across time in the real world, followed by an expositional interview with an experimenter. Using this idiographic method, they revealed detailed summaries of the inner experiences of a given individual over time. Furthermore, in obtaining five scans from three individuals using functional magnetic resonance imaging, a study found that each individual showed distinct profiles of classification accuracy across different brain regions relevant to the content of their task-unrelated thoughts (Hung & Hsieh, 2022). Applying connectome-based predictive modeling on this dataset, Kucyi and colleagues additionally found patterns of functional connectivity that predicted task-unrelated thought within individuals, but these patterns did not generalize to the other two individuals in the study (Kucyi et al., 2024).

These findings highlight the main benefits of an idiographic approach in studying thoughts and how it complements existing group-level findings: It provides a comprehensive and more representative summary of an individual’s thoughts, which in turn increases how predictable these thoughts are within individuals (Shareef—Trudeau et al., 2025). Specifically, since the idiographic approach considers intra-individual variation as a meaningful characteristic of individuals (Molenaar, 2004; Molenaar & Campbell, 2009), it acknowledges the heterogeneity in the data across time within and across individuals. In the context of thoughts, we use the term “*precision experience sampling*” to refer to an idiographic method that involves sampling ongoing thoughts from a given individual across multiple sessions spanning days, weeks or even months, which characterizes ecological momentary assessment broadly, and additionally examining the sampled experiences at the individual level.

The benefits of acquiring a large amount of information about thoughts within individuals are twofold. First, from a theoretical perspective, it furthers our understanding of the idiosyncratic patterns of how thoughts differentially unfold across time as well as mental, emotional or physical states across individuals. Second, from an applied perspective, it allows for more accurate predictions to be made about the thoughts of a given individual. Given the implications of various thought dimensions in medical and clinical conditions, such as Alzheimer’s disease (Gyurkovics et al., 2018), depression (Chaieb et al., 2022) and social anxiety (Arch et al., 2021), the capacity to predict when a specific individual engages in certain thought patterns related to these conditions offers the opportunity to tailor interventions to individuals based on their own data. There are important clinical implications of making these person-specific predictions, especially given the heterogeneity in symptom expression and treatment response in these medical and clinical conditions. These applications of the idiographic approach with precision experience sampling aligns with the notion of precision medicine, which aims to tailor disease prevention and treatment to individuals by accounting for individual differences (Collins & Varmus, 2015). Similar applications have been considered in psychiatry, with the aim to provide personalized treatment strategies to individuals (Manchia et al., 2020) as well as in neuroimaging, with the aim to obtain accurate and reliable maps of neural network organization within individuals (Gordon et al., 2017).

There are several ways in which the idiographic approach with precision experience sampling can inform our understanding of ongoing thoughts that complement existing findings based on the nomothetic approach. For example, empirical evidence has established that the current task plays a role in the types of thoughts that occur at the group level (Chitiz et al., 2023; Mckeown et al., 2025; Mulholland et al., 2023), supporting the Context Regulation Hypothesis suggesting that contextual factors such as task-at-hand can modulate our thoughts and their associated consequences (Smallwood & Andrews-Hanna, 2013). An important extension of these findings is to determine whether the current task differentially predicts ongoing thoughts for different individuals; for example, reading may increase off-task thought in some but decrease off-task thought in others. This also addresses a related question concerning to what extent group-level patterns accurately represent individual cases. In addition, this approach affords the opportunity to examine whether we can make person-specific predictions about the occurrence of a thought dimension based on the task-at-hand using machine learning. In line with past findings that highlight the importance of considering individual and situational factors in making person-specific predictions about future behavior (Beck & Jackson, 2022), this marks an important step toward developing an approach that makes personalized predictions about future ongoing thoughts. Importantly, it has methodological implications for examining individual differences in naturalistic thoughts and clinical implications for person-specific predictions from a precision medicine perspective.

### The current study

The current study therefore used precision experience sampling as an idiographic method to examine individual variations in ongoing thoughts. Specifically, we aimed to 1) establish whether individual differences exist in modulatory effects of task on ongoing thoughts as well as the extent to which group-level patterns correspond to individual-level patterns and 2) determine the utility of this approach in making person-specific predictions of ongoing thoughts based on the task-at-hand using machine learning. To address this, we collected data from two separate groups of participants (representing either the idiographic or nomothetic approach). The *idiographic dataset* was gathered from seven participants, each of whom completed seven experimental sessions. In each session, participants were asked to report on several dimensions of their ongoing thoughts during naturalistic tasks. The *nomothetic dataset* involved collecting data from 49 participants who each completed one experimental session, which primarily served the purpose of validating and comparing patterns from the smaller idiographic group. We hypothesized that a) individuals would show differential effects of task on ongoing thoughts, b) individual-level patterns would not reliably correspond to the group-level patterns, and c) the classification of ongoing thoughts using machine learning would be more accurate in the idiographic group compared to the nomothetic group.

## Methods

### Participants

#### Idiographic dataset

For the idiographic group, we recruited seven individuals (4 females and 3 males; age: *M* = 24.57 years, *S.D.* = 3.95; years of education: *M* = 16.43, *S.D.* = 1.51), who were undergraduate and graduate students. This dataset was part of a larger study that involved other physiological measures collected for these seven participants and has been published elsewhere (Kam et al., 2025; Rahnuma et al., 2024).

#### Nomothetic dataset

In the larger nomothetic group, we recruited 49 individuals (38 females, 9 males, 1 transgender male and 1 non-binary; age: *M* = 19.67, *S.D.* = 1.43; years of education: *M* = 13.41, *S.D.* = 1.17), who were undergraduate students.

#### Idiographic and nomothetic groups

Given the small sample size in the idiographic group, we collected a second dataset based on the same protocol to create a nomothetic group for the purpose of validating the idiographic dataset. The nomothetic dataset was designed in such a way that both groups had the same total number of experimental sessions: The idiographic dataset consisted of seven sessions from a smaller sample (n = 7), whereas the nomothetic group consisted of one session from a larger sample (n = 49). Participants from both groups had normal or corrected-to-normal vision and did not self-report neurological disorders. Idiographic participants received monetary compensation, and nomothetic participants received course credits. All participants provided written informed consent. The procedures in this study adhere to the principles of the Declaration of Helsinki and were approved by the Conjoint Faculties Review Ethics Board at the University of Calgary.

### Experimental procedure

Across both groups, participants were instructed to perform any task they wished on a desktop computer in the same room in our laboratory. During these self-selected tasks, thought probes occasionally occurred that prompted participants to report the nature of their ongoing thoughts. The procedure was identical across groups with the following exceptions. First, idiographic participants completed seven sessions (in line with an idiographic approach), whereas nomothetic participants completed one session (in line with a nomothetic approach). Second, additional measures were collected in idiographic participants (including electroencephalogram and eye tracking), which took approximately an extra 45 to 60 min to prepare prior to data collection. Data from both groups consisted of a total of 49 datasets (idiographic group: seven participants x seven sessions and nomothetic group: 49 participants × 1 session).

### Self-selected tasks and thought probes

The following procedure was identical across both groups. During each session, participants performed any task they wished on the provided computer for approximately 80 min. By allowing participants to select their own tasks, we aimed to increase the ecological validity of the chosen tasks and engage participants in naturalistic activities. We coded their typed response to the self-selected tasks into the following categories: reading/studying, writing/editing, watching videos, browsing or surfing the Internet, playing games (e.g., such as Sudoku or racing games), other more cognitively demanding tasks (e.g., creating PowerPoint presentations, programming) and miscellaneous (e.g., doing nothing). As the proportion of responses in the “miscellaneous” category was very low (0.1%), we did not proceed with this category and data in this category were excluded from subsequent analyses. Our decision to group certain activities together was based on their shared processes or estimated levels of task demands, as well as pragmatic concerns about levels of variables to be included in the analyses. For example, we combined reading and studying into one category because both involved reading comprehension; we also combined writing and editing into one category because both involved recursive or iterative processes of constructing phrases or sentences. For the other more cognitively demanding tasks category, we included any activity that appears to be cognitively demanding and thus grouped them based on estimated cognitive demands (though this was not verified by the participants) which also contrasts with the miscellaneous category that involve responses such as “doing nothing.” Finally, we combined certain activities into one category in order to ensure there were sufficient occurrences for each task category and to avoid excessive numbers of levels in the “task” variable.

Throughout the self-selected tasks, thought probes were presented approximately every 2 min, ranging from 1.5 to 2.5 min to minimize expectation effects (Gouraud et al., 2021; Jin et al., 2019, 2020). These probes presented a list of questions that appeared on top of all other activities on the monitor, accompanied with a tone (1000 Hz, 200 ms) alerting participants of its occurrence. Each multi-dimensional experience sampling thought probe prompted participants to respond to questions about several dimensions of their ongoing experience immediately preceding the probe. First, they briefly reported their self-selected task, defined as the activity they currently aimed to do. This enabled us to examine whether the types of tasks being performed differentially impact ongoing thoughts. Following this, participants characterized their ongoing thoughts by responding to questions about their thoughts in terms of content (i.e., internally oriented, off-task, self and others-oriented), dynamics (i.e., freely moving, goal-oriented, sticky) and form (i.e., visual and auditory) on 7-point Likert scales (as reported in Supplementary Table S1). This ranged from 1 = not at all to 7 = extremely for all questions except for internally oriented thoughts (1 = internally oriented to 7 = externally oriented) and off-task thoughts (1 = on-task to 7 = off-task). These are some of the most common questions included in studies of ongoing thoughts (Mckeown et al., 2021; Mulholland et al., 2023; Wang et al., 2018) and spontaneous thoughts (Mills et al., 2018, 2021). Our choice of these thought dimensions was based on several factors, including their prevalence, their impact on daily life and clinical implications and their potential to show reliable physiological markers (as a part of the research aims of a larger study). By inquiring about multiple dimensions of ongoing thoughts at each thought probe, we obtained a more comprehensive summary of individuals’ ongoing thoughts. At the beginning of the study, we provided definitions and example scenarios of each question to ensure participants understood the meaning of these questions (as reported in Supplementary Table S1). We presented 35 thought probes throughout each session for both studies. In the idiographic group, each of the seven participants was presented with a total of 35 thought probes × 7 sessions = 245 thought probes. The adoption of precision experience sampling allows us to examine the effects of task on thoughts in each of the seven participants in a person-specific manner. In the nomothetic group, each of the 49 participants were presented with a total of 35 thought probes. Both studies consisted of a total of 1715 probe-level data points (idiographic group: 245 probes × 7 participants; nomothetic group: 35 probes × 49 participants).

### Statistical and machine learning analyses

We have two primary objectives. Our first aim concerns individual differences in the modulatory effects of task on ongoing thoughts as well as the correspondence between individual- and group-level patterns. To address this, we examined whether task-at-hand differentially predicted these dimensions of ongoing thoughts using linear mixed effects models in the idiographic group. We implemented these analyses at the individual and group level, which we elaborate below. Our second aim concerns whether the accuracy of predicting ongoing thoughts based on the task-at-hand is enhanced in the idiographic group with precision experience sampling. For this, we implemented machine learning analyses to make person-specific predictions of ongoing thoughts for each participant in the idiographic group and compared their classification performance to those in the nomothetic group.

#### Descriptives and reliability of assessment of ongoing thoughts

Prior to addressing the primary research questions, we examined descriptive measures to characterize the dimensions of ongoing thoughts in individuals in the idiographic group. Given the smaller sample in the idiographic group (n = 7), we compared these measures to a larger sample in the nomothetic group (n = 49), as a way to verify that the reports of thoughts in the idiographic group are generalizable beyond the seven participants in this group. This includes comparing the means and variability of thought dimension ratings, the relationship between thought dimensions as well as the reliability of the assessment of ongoing thoughts.

#### Is the occurrence of thought dimensions comparable between groups?

To examine whether the mean ratings of dimensions of ongoing thoughts were similar across the idiographic and nomothetic groups, we implemented separate linear mixed effects model analyses to compare the two groups on each of the nine dimensions. Each model included group as a fixed effect and participant as a random intercept when predicting the rating of a thought dimension at the probe level. Significance of a main effect was determined by comparing models with and without the predictor of interest (i.e., group) using likelihood ratio tests. Participants in the two studies differed in age (*t* = 3.25, *p* =.017) and years of education (*t* = 5.07, *p* =.001), but there is no evidence of differences in gender ($\chi$ ^*2*^ = 2.34, *p* =.505, corroborated by the Bayesian contingency table analysis with BF_10_ = 0.07 which can be interpreted as no gender difference between studies). Given that age and years of education were highly correlated (*r* = 0.87 in the idiographic group and *r* = 0.86 in nomothetic group), we only included age as a covariate to avoid issues of multi-collinearity in these analyses.

We also implemented similar analyses to compare the standard deviation in thought dimension ratings, which was computed across probes within an experimental session separately for individuals in each group. Each model included group as a fixed effects predictor, participant as a random intercept and age as a covariate, in predicting the standard deviation in ratings of a given thought dimension computed at the session level.

Given that the number of completed tasks throughout a session may impact the occurrence and variability of thoughts, we also included the number of unique task categories reported as a covariate in the above models. As the inclusion of this covariate did not change the significance or direction of results in any of these analyses, we report these results in Supplementary Table S3. The final models reported below did not include this covariate.

#### Are the relationships between thought dimensions similar between groups?

Beyond the occurrence of a given thought dimension, we compared the associations between thought dimensions across the idiographic and nomothetic groups. First, we compared the grand average correlation matrices between groups, which captures the overall relationship between each pair of thought dimensions across participants. To do this, we computed a correlation matrix that included the ratings of all nine thought dimensions during each session and averaged the correlation matrices across all 49 sessions separately for each group. We compared these two grand average correlation matrices for the idiographic and nomothetic groups using the mantel test, which is similar to a correlation test designed to test the similarity of two data matrices.

#### Is the reliability of ongoing thought measures comparable between groups?

Next, we determined whether the reliability of these measures of ongoing thoughts differed across groups. Specifically, we computed intraclass correlation coefficients as an index of reliability. Since we measured ongoing thoughts that are naturally fluctuating moment to moment in the real world (which provides more opportunities for spontaneous fluctuations in ongoing thoughts), we anticipated that the intraclass correlation coefficient values in our study would be lower compared to other methodological approaches. The intraclass correlation coefficient represents the proportion of total variance that is attributable to between participant variance. The responses at each thought probe for each participant served as inputs. In particular, the ratings at each thought probe for the idiographic group were nested within sessions within participants, whereas the ratings were nested within participants for the nomothetic group. Accordingly, the intraclass correlation coefficients were estimated based on differing numbers of measures within participants in both groups. The idiographic group and nomothetic group were designed to provide a differing amount of information at the individual level, with a total of 245 data points (35 probes × 7 sessions) for each of the seven participants in the idiographic group and a total of 35 data points for each of the 49 participants in the nomothetic group. To examine whether having more data points per participant in the idiographic group would change the reliability of ongoing thought assessments, we compared the intraclass correlation coefficients of each of the ongoing thought dimensions between groups.

In summary, these three sets of preliminary analyses on the occurrence of thought dimensions, relationships between thought dimensions and reliability of assessments served the purpose of validating the data of ongoing thoughts reported by participants in the idiographic group.

#### Predictive effects of task on ongoing thoughts

*Linear mixed effects models*. Next, we addressed two primary research questions. Our first aim was to determine whether there are individual differences in the modulatory effects of task on ongoing thoughts, and whether these individual patterns correspond to group-level patterns. These analyses would inform whether individual differences exist in the task-related effects on ongoing thoughts, and whether general claims about the relationship between tasks and thoughts reliably represent individual patterns. Leveraging the increased data points obtained from each participant through precision experience sampling in the idiographic group, which provided sufficient data to examine these effects at the individual level, we only implemented this set of primary analyses within individuals in the idiographic group. Furthermore, we also implemented the group-level analyses in the nomothetic group as it allows us to determine whether the effects of task on thought dimensions are also observed in a larger sample, which points to the generalizability of the idiographic data. In general, the main effect of task was significant for all thought dimensions (except for one) in the nomothetic group. These results are reported in more detail in Supplementary Table S17.

To address this first aim, we first determined whether the task-at-hand differentially predicted ongoing thoughts by implementing separate linear mixed effects model analyses for each of the nine thought dimensions in the idiographic group. Prior to analysis, the ratings of the nine thought dimensions were centered around each participant’s own mean (MacVittie et al., 2024a; b). We then implemented these analyses at two levels: individual level and group level. These analyses were first implemented at the individual level for each participant to determine whether they showed distinct patterns of task-modulatory effects on thought dimensions. To test this, each model included task as a fixed effects predictor, as well as session as a random intercept, in predicting the rating of a given thought dimension at the probe level. We applied a Bonferroni correction for multiple comparisons for the nine thought dimensions (such that $\alpha$=.05/9 =.006) since our primary research aim involves statistical inferences regarding whether task modulates each thought dimension. We did not additionally correct for comparisons across individual participants as our study was less concerned about making statistical inferences about any particular participant. However, we note in Table 1 when a statistical result at the individual level reached a more conservative Bonferroni-corrected threshold accounting for all participant (7) by thought dimension (9) comparisons ($\alpha$= 0.05/63 =.0008). If the main effect of task was significant, we implemented pairwise comparisons between tasks (with false discovery rate correction for multiple comparisons) to examine their effects on thought dimension ratings as follow-up analyses to examine which tasks specifically modulated a given thought dimension. Next, we implemented the analyses at the group level across all seven participants in the idiographic group to determine the extent to which group-level patterns reliably represent individual cases. The group-level analysis was identical to the individual analyses with the additional inclusion of a random intercept of participant nested within session.

Given that the number of tasks completed throughout a session may impact the momentary relationship between ongoing task and thoughts, we also included the number of unique task categories reported as a covariate in the above models. As the significance or direction of results across models remained largely the same after the inclusion of this covariate, we report these results in Supplementary Table S7. The final models reported below did not include this covariate.

*Machine learning analyses*. Our second aim was to establish whether the precision experience sampling idiographic approach enhances within-participant classification accuracy of ongoing thoughts based on the current task. Specifically, we first examined whether person-specific prediction (i.e., using a participant’s data to predict their own ongoing thoughts) is more accurate in the idiographic compared to the nomothetic group. To assess the generalizability of the model in the idiographic group, we additionally compared the person-specific (within-participant) predictions and across-participant predictions (i.e., using group data to predict the ongoing thoughts of a given participant). Similar to the objective above concerning whether group-level patterns represent individual patterns, this assesses whether the accuracy of prediction of ongoing thought is higher when using one’s own data versus group data. Notably, this comparison may highlight not only the benefits of using one’s own data over group data in predicting ongoing thoughts, but also that this benefit is gained through a relatively small amount of data (i.e., seven sessions in a participant). Overall, this second aim would establish the value of the precision experience sampling idiographic approach, such that it enhances the accuracy of predictions of a given individual’s ongoing thoughts based on that individual’s data, suggesting that this approach is more powerful in making person-specific predictions.

To address this second aim, we first implemented machine learning to determine whether each thought dimension can be predicted by the task-at-hand with higher accuracy in the idiographic group compared to the nomothetic group. We performed within-participant classification separately for each participant in each group. To classify each thought dimension using task-at-hand as an input, we used the k-nearest neighbor (k-NN) classifier, which discriminates a data point based on the majority vote of its neighbors. This is a relatively simple algorithm that is suitable for our dataset compared to more complex models, because it is easy to implement and efficient for smaller datasets (such as ours) and makes no assumption about the data distribution. Importantly, our goal was not to identify the best algorithm for prediction but rather to implement a simple, well-established algorithm that serves the purpose of facilitating comparisons between the two groups. We used the Euclidean distance to find the neighbors. Through the trial-and-error method by assessing a different number of neighbors (e.g., k = 5, 10, 15, 20, 25 and 30), we found that k = 25 led to the best performance and therefore set the number of neighbors (k) as 25. For both groups, each data point consisted of an input feature of task-at-hand (reading/studying, writing/editing, watching videos, web surfing, playing games, other activities) and the output refers to a rating on the 7-point Likert scale of a given thought dimension. To capture the specificity of information offered by the full range of the scale, we used the k-NN classifier to predict one of the seven potential ratings on the 7-point Likert scale for each thought dimension.

To perform the multi-class classification, we used the stratified k-fold within-subject strategy with k-fold set as 3. Since each participant had a smaller number of data points in the nomothetic group, we set the k-fold value as 3 to ensure there are sufficient data points for each class in the test set for each participant, and to maintain uniformity with the classification approach across groups. Moreover, we only selected a type of task if it had a corresponding minimum of 6 data points for a given participant.

We first split the dataset into three folds; then, in each round of classification, one fold will act as test set and the other two folds (k-1) will act as training set. We then computed the average classification performance across all rounds of classification for each participant. We repeated this process 100 times in order to increase the reliability of the obtained classification performance, as well as to perform statistical tests to compare between groups. For each iteration, we randomly split data points into each fold in k-fold cross-validation, which exposes the model to a different training and test sets therefore resulting in slightly different classification performance for each iteration. The implementation of iterations ensures that the reported classification performance is not simply due to the random selection of data points from one iteration and provides a more reliable estimate of classification performance. The mean across all 100 iterations and participants was then calculated and reported as the overall average classification performance for a given thought dimension. Finally, we averaged the classification performance across all seven participants for each thought dimension and compared them across all 100 iterations between the idiographic group and nomothetic group using the Wilcoxon rank sum test (i.e., the nonparametric version of the independent samples t test). Since the datasets were highly imbalanced, the synthetic minority over-sampling technique (SMOTE) (Chawla et al., 2002) was implemented to balance the training datasets and the classifier learned using the balanced train set was used to classify the imbalanced test set. The SMOTE technique is commonly used to address data imbalance in the training set in machine learning. Although sub-sampling in the condition with more data points is more common in some context, we adopted this technique for the machine learning analyses as it better aligns with machine learning practices. Since the test dataset was also highly imbalanced, we used two performance metrics that are insensitive to class imbalance in the test set: Mathew’s correlation coefficient (MCC), which is one of the gold standard metrics for unbalanced data (ranging from − 1 to poor performance to 1 perfect performance, with 0 indicating chance-level performance) as well as balanced accuracy (ranging from − 1 to poor performance to 1 perfect performance).

Given that the idiographic group was designed to have more data points per participant compared to the nomothetic group, this comparison between studies informs whether a larger number of data points per participant (idiographic group) leads to better classification performance compared to less data points per participant (nomothetic group). To determine whether the source of the data points matter in classification performance (in terms of whether they were real data or simulated data), we up-sampled the number of data points per participant in the nomothetic group to match the number of data points per participant in the idiographic group. Specifically, we used the SMOTE technique to up-sample the nomothetic dataset by 7 times to obtain the 245 data points for each participant. We then implemented the identical classification approach described above on this augmented dataset in the nomothetic group. We implemented the Wilcoxon rank sum test to statistically compare the classification performance across all 100 iterations between the idiographic group and this augmented data set in the nomothetic group.

Finally, after establishing the classification performance of the person-specific model, we then tested model generalizability by examining across-participant classification in the idiographic group. This informs whether predictions of ongoing thoughts using the precision experience sampling approach based on group-level data benefits classification performance.

### Data and analysis code

Statistical analyses were performed using R 4.2.2 (R Core Team, 2022) in R Studio (RStudio Team, 2022.12.0 + 353) and the following packages: plyr and dplyr to organize the data; vegan for comparing matrices; psych for intraclass correlation coefficient analysis; lme4, emmeans and car for statistical analysis and model evaluation; and ggplot2 and colorspace for plotting. Machine learning analyses were performed using fitcknn in MATLAB 2021b. This study’s design and its analysis were not pre-registered. All data and analysis code will be available upon request.

## Results

As preliminary analyses, we compared descriptive measures of ongoing thoughts, as well as the reliability of these assessments between the idiographic and nomothetic groups, in order to determine the generalizability of the idiographic dataset. Following this, we implemented two sets of primary analyses to examine ongoing thoughts as a function of the task-at-hand in these seven participants. First, we implemented linear mixed effects analyses at two levels: at the individual level for each of the seven participants to determine the extent to which these patterns are unique to each individual as well as at the group level to determine whether individual-level patterns correspond to group-level patterns. Second, we implemented machine learning analyses to assess the classification performance of ongoing thoughts based on the task-at-hand within the seven participants and compare their classification performance to those in the nomothetic group.

### Descriptives and reliability of assessment of ongoing thoughts

Prior to addressing the primary research question, we first sought to examine the thought ratings in the idiographic group by comparing their descriptives on thought dimensions, the relationships between them, as well as the reliability of the assessment of ongoing thoughts to the nomothetic group.

#### Comparison of descriptives of thought dimensions between groups

**Fig. 1:**
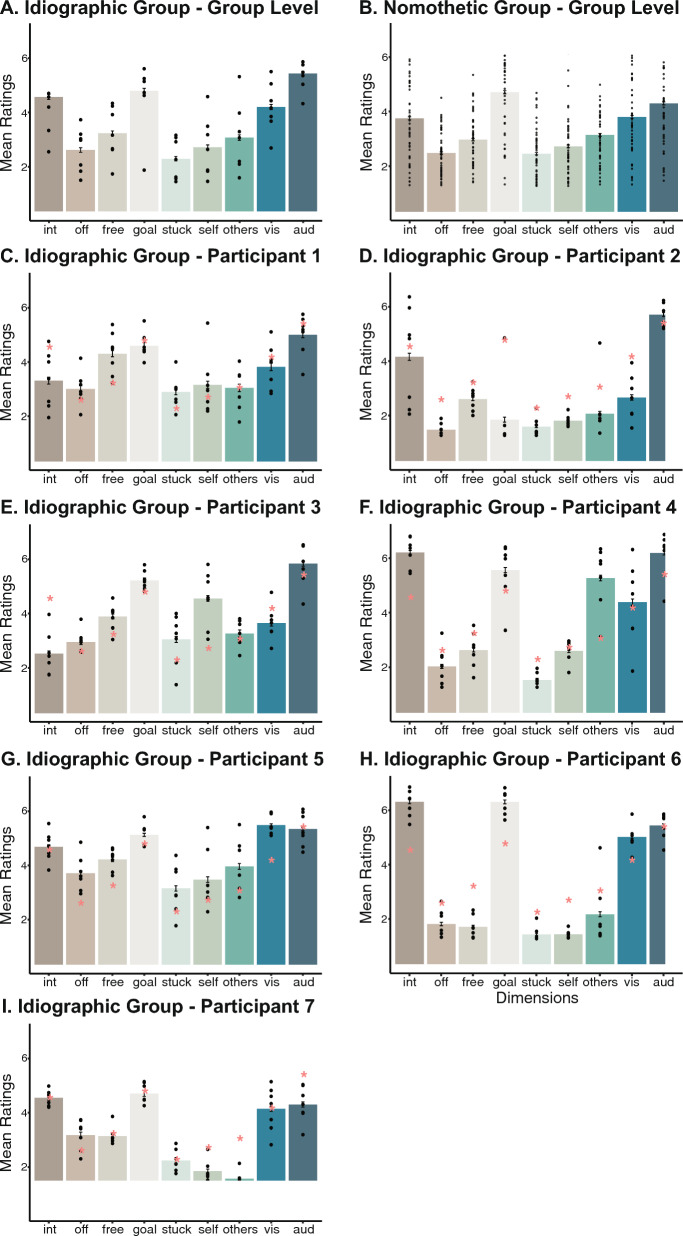
Overall thought dimension ratings at the group level in the idiographic and nomothetic groups and at the individual level in the idiographic group. *Note:* The mean ratings were averaged across participants in the idiographic group **A** and nomothetic group **B** and averaged across sessions within participants for each of the seven participants **C**–**I** in the idiographic group. Each black dot represents the mean rating of an individual for group-level plots **A**–**B** and the mean rating in a single session for individual-level plots **C**–**I**. The red asterisks in the individual plots represent the mean rating (based on data shown in **A**) in the idiographic group for ease of comparison between individual- and group-level ratings. The ratings ranged from 1 = not at all to 7 = extremely for all thought dimensions except for internally oriented thoughts (1 = internally oriented to 7 = externally oriented) and off-task thoughts (1 = on-task to 7 = off-task). Int = internally oriented; off = off-task; vis = visual; and aud = auditory

First, we compared the overall mean ratings of each of the nine thought dimensions across the idiographic and nomothetic groups. Seven of the dimensions differed in the mean ratings between studies. Specifically, the idiographic participants reported having significantly higher levels of off-task thought, freely moving thought, goal-oriented thought, sticky thought, self-oriented and auditory thought, as well as lower levels of others-oriented thought than the nomothetic participants (all *p* <.001). There were no significant differences between groups in the mean ratings of the internally oriented (*p* =.137) and visual modality (*p* =.010) thought dimensions. The mean ratings of each of the nine thought dimensions for the two groups are depicted in Fig. 1.

Next, we compared the variability of the ratings of the nine thought dimensions between studies by examining the standard deviation of ratings in the idiographic and nomothetic groups. Although the standard deviation in the ratings was generally higher in the nomothetic participants compared to the idiographic participants for all nine thought dimensions, only the comparison for sticky thought was statistically significant (*p* =.026). This indicates the variability of the thought dimension ratings is similar between groups. Details of both sets of descriptive analyses are reported in Supplementary Table S2.

In addition to group-level data, we also present the mean and standard deviation of the individual-level ratings of each of the seven participants in the idiographic group in Fig. 1 as well as Supplementary Figure S1. Figure 1 highlights both inter-individual and intra-individual variability. In particular, although some participants are more similar to each other (e.g., Participants 5 and 7) in the relative ratings of thought dimensions, the absolute and relative ratings of the nine thought dimensions generally differed across the seven participants. For instance, Participant 2 reported low levels of goal-oriented thoughts among all thought dimensions, whereas Participant 6 reported highest levels. However, the auditory thought dimension was consistently rated at high levels across participants. Moreover, there is also variability across sessions within participants. As an example, Participant 2 reported a widespread range from 2 to 6 on a 7-point Likert scale as a session-level mean rating on the internally oriented thought dimension across the seven sessions. We illustrate this intra-individual variability in showing the probe-level data for all seven sessions for an exemplar participant in Supplementary Figure S2.

To further explore different patterns at the individual level, we also report the descriptives of the tasks engaged by each of the seven participants in the idiographic group in Supplementary Table S5. For example, Participants 1 and 6 reported most instances of reading, yet they reported differing levels on most dimensions of thoughts when reading (as shown in Fig. 3). Similarly, Participants 3 and 5 reported most instances of playing games, yet they reported differing levels of thoughts for several dimensions. In addition to individual variability observed at the thought level, these descriptives further illustrate the intra-individual differences in how task modulates thoughts. We formally test these observations below.

#### Comparison of thought dimension relationships between groups

**Fig. 2:**
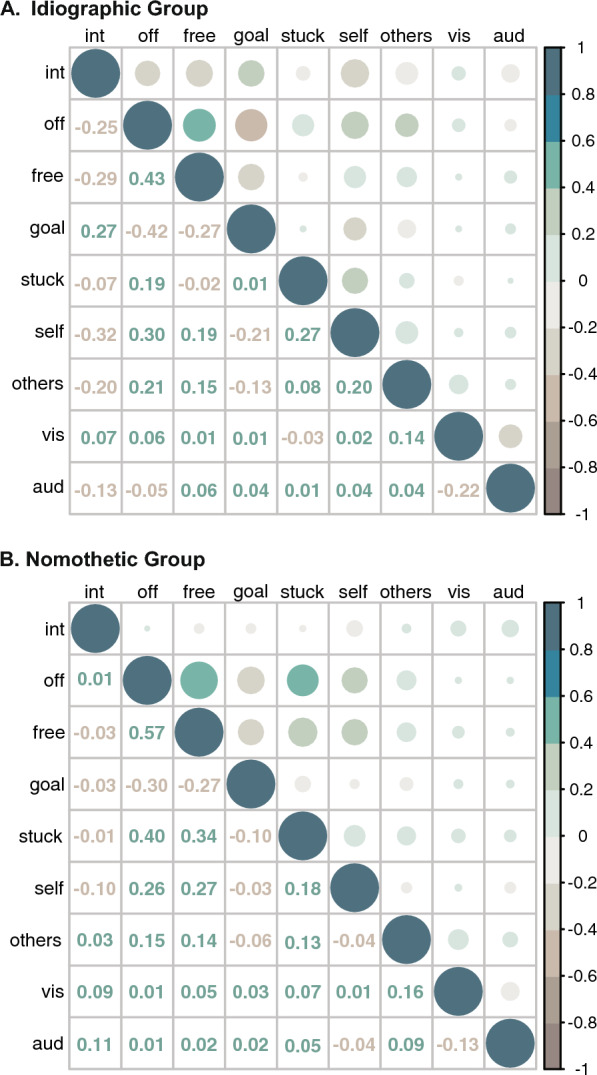
Correlation matrices of thought dimensions in the idiographic and nomothetic groups. *Note:* The correlations between thought dimensions are shown for the idiographic group A and nomothetic group B. The lower diagonal presents the exact correlation value. In the upper diagonal, the size of the circles represents the magnitude of the correlation, whereas the color represents the direction of the correlation. The upper diagonal facilitates comparison between the two groups, illustrating that the direction of the correlation between thought dimensions is comparable across groups. Int: internally oriented; off: off-task; vis: visual; and aud: auditory

To assess whether the relationship between thought dimensions was similar between the idiographic and nomothetic groups, we implemented comparisons at the group level based on the grand average correlation matrices. Comparison of the correlation matrices of the two groups revealed they were significantly correlated (*r* = 0.63, *p* =.003). Specifically, although there is variation in the magnitude of the correlation between a given pair of thought dimensions across groups, the direction of the correlations is consistent between them. This suggests the overall patterns of correlations between thought dimensions are similar between groups. We also observed correlations between established thought dimensions that are consistent with previous literature and theoretical frameworks. For example, task-unrelated thought was positively correlated with freely moving thought (idiographic: *r* = 0.43; nomothetic: *r* = 0.57; Mills et al., 2018; Thiemann et al., 2023); freely moving thought was negatively correlated with goal-oriented thought (idiographic: *r* = − 0.27; nomothetic: *r* = − 0.27; (Christoff et al., 2016). Given these observations corroborate past findings, it further serves as validation of our experience sampling data in the idiographic as well as nomothetic group. The correlation matrices for both groups are presented in Fig. 2.

#### Comparison of reliability of ongoing thought measures between groups

Next, we determined whether the reliability of measures of ongoing thoughts differed across the idiographic and nomothetic groups. The intraclass correlation coefficient revealed that the overall reliability across thought dimensions was numerically higher in the idiographic group compared to the nomothetic group (idiographic: *M* = 0.47, *SD* = 0.10; nomothetic: *M* = 0.37, *SD* = 0.10). Given ongoing thoughts naturally fluctuate moment to moment, with variability likely to increase in the real world, this aligns with our expectation that reliability would be lower using our measure relative to other approaches.

In comparing each thought dimension, we found that reliability was numerically higher in the idiographic group for approximately half of the thought dimensions compared to the nomothetic group, including the internally oriented, off-task, freely moving, goal-oriented, self-oriented and others-oriented dimensions. The opposite pattern was observed for the auditory thought dimension, such that its reliability was higher in the nomothetic group compared to the idiographic group. Sticky and visual thought dimensions showed comparable values. Together, this indicates that having more data points within individuals in the idiographic group increased reliability for several thought dimensions that broadly capture the content (i.e., off-task, self- and others-oriented) and dynamics (i.e., freely moving, goal-oriented) of thoughts, suggesting that these dimensions are more consistent across time and context. In contrast, having less data within individuals but more data at the group level in the nomothetic group increased reliability for thought dimensions that mainly capture the modality of thoughts (i.e., auditory modality), suggesting that this dimension shows more variable ways in which an individual’s thoughts unfold over time and across context. These results are reported in Supplementary Table S6.

Collectively, these preliminary analyses indicate that although some differences emerged in the mean levels as well as reliability (as indexed by ICC) of thought dimension ratings, the variability of thought dimensions in the idiographic group was similar to those in the nomothetic group.

### Predictive effects of task on ongoing thoughts

#### Examining task effects on thought dimensions using linear mixed effects analyses

**Fig. 3:**
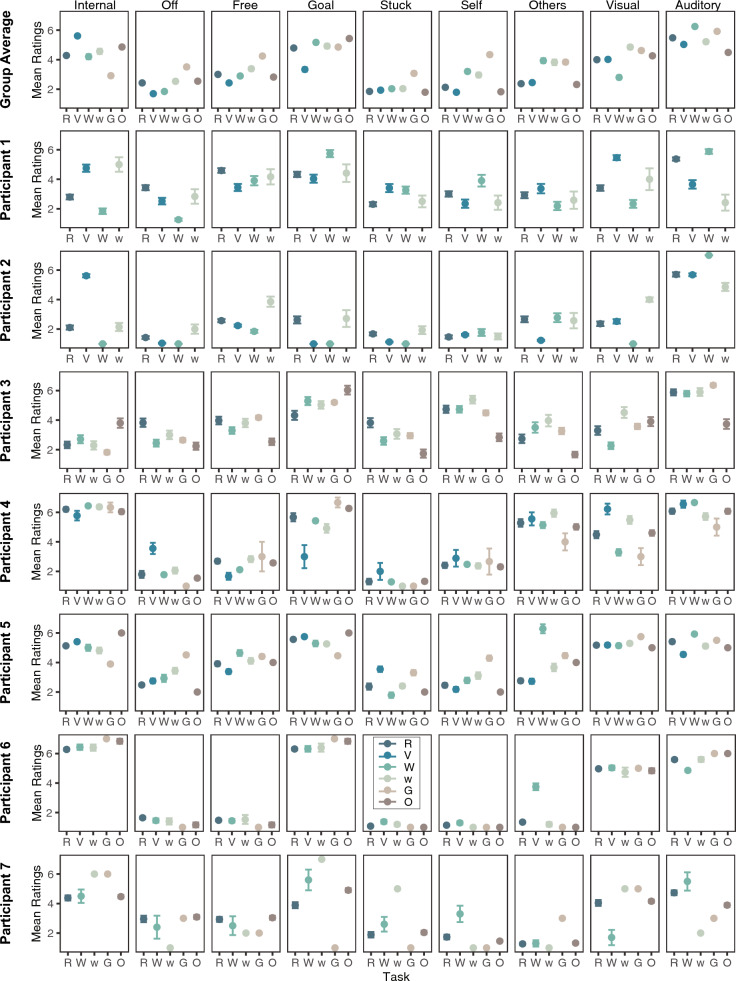
Descriptives of thought dimension ratings as a function of task in the idiographic group at the group level and individual level. *Note:* The mean ratings of all nine thought dimensions are presented as a function of the current task at the group level as well as individual level for each of the seven participants in the idiographic group. R: reading/studying; V: video watching; W: writing; w: web surfing; G: playing games; and O: others

To address the first aim, we leveraged the multiple sessions of data available for the seven participants in the idiographic group and examined whether the task-at-hand differentially predicts ongoing thoughts across participants. Specifically, we implemented linear mixed effects model analyses at the individual level for each of the seven participants in the idiographic group. There were notable individual differences in whether task significantly modulated a given thought dimension across participants. For example, for the off-task and visual thought dimension, five participants had a significant main effect of task. In contrast, for the sticky thought dimension, only one participant had a significant task effect. For all other thought dimensions, the number of participants who showed a significant main effect of task ranged from two to five. This underscores individual differences in whether task modulated a given thought dimension across our seven participants. The results of these individual-level analyses are summarized in Table 1. The mean ratings of each thought dimension as a function of task for each participant are shown in Fig. 3.

**Table 1:** Main effect of task on thought dimension ratings in each participant and across participants in the idiographic group

Thought dimension	P1	P2	P3	P4	P5	P6	P7	Group
Internal–External	34.81 ***	86.17 ***	10.38 *	10.60	45.98 ***	1.33	2.65	75.68 ***
Task-relatedness	46.63 ***	30.41 ***	21.45 ***	16.46 **	96.24 ***	1.98	7.82	82.03 ***
Freely moving	5.44	26.93 ***	27.92 ***	16.48 **	31.82 ***	5.14	1.65	53.74 ***
Goal-oriented	23.05 ***	11.76 *	21.75 ***	7.59	64.54 ***	1.42	26.64 ***	57.67 ***
Sticky	10.76 *	21.37 ***	8.52	8.71	7.02	3.22	5.62	7.96
Self-oriented	1.84	1.23	10.30 *	2.42	48.93 ***	3.81	20.01 ***	18.33 **
Others-oriented	5.65	39.41 ***	22.13 ***	9.31	71.64 ***	72.50 ***	6.72	27.10 ***
Visual modality	57.93 ***	43.24 ***	29.30 ***	15.56 *	22.78 ***	7.13	21.69 ***	140.71 ***
Auditory modality	89.78 ***	40.16 ***	39.11 ***	10.09	2.53	3.11	8.92	97.20 ***

This reports the $\upchi$ ^2^ values and their corresponding significance (*** significant after Bonferroni correction for multiple comparisons across nine thought dimensions and seven participants for the individual-level analyses at p <.0008, ** significant after Bonferroni correction for multiple comparisons across nine thought dimensions at p <.006, * significant without correction for multiple comparison at p <.05) based on likelihood ratio tests using linear mixed effects analyses examining the main effect of task in predicting a thought dimension for each participant (P1 to P7) and across participants (at the group level) in the idiographic group (as shown above in separate columns)

Interestingly, the pairwise contrasts between types of tasks in the post hoc comparison also revealed different patterns for each participant. For instance, Participant 2 reported highest levels of off-task thought during web surfing compared to all other activities, whereas Participant 6 reported similar levels of off-task thought across all reported activities. Moreover, for a given pairwise comparison, there were no instances in which all participants showed the same effect. As an example, the highest number of participants who reported similar effects were four participants who reported higher levels of visual thoughts during web surfing compared to writing or higher levels of off-task thoughts during reading compared to writing; for all other pairwise comparisons between tasks, only one or two participants showed a significant contrast. These analyses indicate that the specific tasks that modulate ongoing thoughts vary widely across individuals. The results of these pairwise comparisons at the individual level are summarized in Table 2 and reported in detail in Supplementary Tables S8–16.

**Table 2:** Pairwise contrasts as follow-up to significant task effect on thought dimension ratings predicted in the idiographic group at the individual and group level

Pairwise Contrasts	Int-Ext (P1,P2,P3*,P5, group)	Task (P1,P2,P3,P4, P5,group)	Free (P2,P3,P4,P5, group)	Goal (P1,P2*,P3,P5, P7,group)	Sticky (P1*,P2)	Self (P3*,P5,P7, group)	Others (P2,P3,P5,P6, group)	Visual (P1,P2,P3,P4*, P5,P7, group)	Aud (P1,P2,P3, group)
Re-Vi	P1*** P2*** Gp***	P2** Gp*	P4* Gp**	P2*	P2**		P6*** Gp*	P1*** P2*** Gp***	P1*** P2*** Gp***
Re-Wr	P1** Gp*	P1*** P2* P3** P4* Gp***	Gp*	P1*** P2* P3* P7** Gp***	P1* P2*	P7*	P2*** P5*** Gp**	P1*** Gp***	P1** Gp**
Re-We	P1*** Gp*	P2** P3* P5***	P2***			P5**	P3* P5*** Gp*	P2** P3** Gp***	P1*** Gp***
Re-Ga	P5*** Gp*	P3** P5***	P5*** Gp***	P3* P5***		P5*** Gp*	P5*** Gp*		
Re-Ot		P3** Gp**		P3*** P7** Gp***				P5*** P7** Gp*	P3*** Gp**
Vi-Wr	P1*** P2*** Gp***	P1*** P4* Gp***	P4*	P1*** Gp***			P2*** P5***	P1*** P2*** Gp***	P1*** P2*** Gp***
Vi-We	P2*** Gp***	P2*** P5*	P2*** P4** Gp***	Gp*	P2**		P6***	P1** Gp*	P2** Gp*
Vi-Ga	P5*** Gp***	P5*** Gp**	P4* P5*** Gp***	P5***	P2*	P5**	P5* P6**	Gp***	Gp***
Vi-Ot	Gp***		P4**	Gp***			P6*** Gp*	Gp***	Gp**
Wr-We	P1*** Gp***	P1** P2*** Gp***	P2*** Gp**	P1* P3* Gp**		P5**	P2*** P5*	P1** P2** P3*** P4* Gp***	P1*** P2* Gp***
Wr-Ga	P5**	P5*** Gp***	P3* Gp***	P5** P7* Gp***		P5***	P5*	P5** Gp***	
Wr-Ot	Gp*	Gp**				P7** Gp*	P3** Gp**	P3** P7** Gp***	P3*** Gp***
We-Ga	P5** Gp***	P5** Gp**	Gp*	P5**				P3** Gp***	Gp***
We-Ot			P3** Gp**	P3* Gp**			P3** Gp*		P3***
Ga-Ot	P3* Gp**	Gp***	P3*** Gp***	P3* Gp***		Gp**	P3* Gp*		P3*** Gp**

This table reports significant pairwise contrasts for a given pair of tasks at the individual-level (participants P1-P7) as well as group-level effects (Gp) in the idiographic group and their corresponding FDR corrected significance level (*** p <.001, ** p <.01, * p <.05) separately for each thought dimension. In the first row, those who showed a significant main effect of task for that thought dimension are listed in brackets (with those with asterisks denoting significance that did not survive correction for multiple comparison as reported in Table 1). Each row shows each pairwise comparison across the six task categories, with the first task in each pair in the column heading as the reference. Positive effects are indicated by an underline, and negative effects are shown in regular font style; for example, Participant 1 showed significantly higher levels of auditory thoughts during reading compared to video watching and lower levels of auditory thoughts during reading compared to writing. Re = reading/studying; Vi = watching videos; Wr = writing; We = web surfing; Ga = games; and Ot = other cognitively demanding tasks. Int = internally oriented thought; Ext = externally oriented thought; and Aud = auditory thoughts. A more detailed report of these analyses for each thought dimension is reported in Supplementary Tables S8–16

We then examined the effects of task on thoughts at the group level across the seven participants to assess whether the individual patterns reported above correspond to group-level patterns in the idiographic group. There was a significant main effect of task at the group level across dimensions (*p*’s <.001), with the exception of the sticky (*p* =.158) thought dimension. These group-level effects appear to represent patterns observed at the individual level for some thought dimensions but not others. As an example, the majority of participants (n = 5) showed a significant effect for off-task thoughts, corresponding to the group-level effect. On the other hand, only three participants (of which one did not survive multiple comparison correction) showed a significant effect of task on self-oriented thought, yet there was a significant effect at the group level. These results are reported in Table 1.

Pairwise contrasts between tasks in the post hoc comparison also revealed differing results at the group level compared to the individual level. In some cases, significant effects at the group level were not observed in most participants, and the non-significant effects at the group level were observed in many participants at the individual level. For instance, writing predicted significantly lower levels of off-task thought than surfing the web, playing games and other activities at the group level. However, this was only observed in a small sample of participants at the individual level (e.g., n = 1 in comparing writing and play games and n = 2 in comparing writing and web surfing).

The patterns of results for the other thought dimensions also converge on the observation that types of tasks differentially predicted each thought dimension across the group and individual levels. These analyses indicate that the overall patterns of the modulation of task-at-hand on ongoing thoughts observed at the group level do not necessarily generalize well to individual participants. These results are summarized in Table 2 and reported in more detail in Supplementary Tables S8–16.

#### Predicting thought dimensions using machine learning analyses

Having established individual differences in the modulatory effects of task on a given thought dimension, we then examined whether the precision experience sampling idiographic approach leads to more accurate person-specific predictions (i.e., predicting an individual’s ongoing thought with their own data). To address this second aim, we first implemented machine learning analyses to determine whether a thought dimension can be predicted by task-at-hand with higher accuracy within individuals in the idiographic group compared to the nomothetic group. The classification performance using the k-fold within-subject strategy was superior for all thought dimensions in the idiographic group relative to the nomothetic group. For example, all thought dimensions achieved decent classification performance (MCC, *M*s = 0.21 to 0.38). The nomothetic group without data augmentation mainly attained below chance-level performance (MCC = 0).

Since the idiographic group was designed to have more data points per subject compared to the nomothetic group, this finding indicates better classification performance with a larger compared to smaller number of data points per participant. Notably, to verify that the difference in classification performance was not simply due to the number of data points available per participant, we up-sampled the number of data points for each participant in the nomothetic group to equate the number of data points per participant in the idiographic group. The thought dimensions in the nomothetic group with data augmentation attained classification performance that were slightly above chance (MCC, *M*s = 0.03 to 0.12). Although augmenting the data in the nomothetic group enhanced classification performance, the idiographic group still attained better classification performance. Specifically, results from the Wilcoxon rank sum tests revealed that the idiographic group showed superior classification performance on all thought dimensions, as compared to the nomothetic group without data augmentation (all *p’*s <.001) and with data augmentation (all *p’*s <.05). These findings indicate enhanced accuracy in person-specific predictions of ongoing thoughts with the precision experience sampling idiographic approach compared to the nomothetic approach. This pattern remained the same, even after matching the number of data points across groups, highlighting the value of obtaining a large amount of real data within participants (compared to using augmented data). The results of these analyses are reported in Table 3.

**Table 3:** Comparison of within-participant classification performance of task predicting ongoing thoughts between idiographic and nomothetic groups

Thought	Idiographic *(original)*		Nomothetic *(original)*		Nomothetic *(up-sampled)*	
Dimension	MCC	BA	MCC	BA	MCC	BA
Internal–External	0.29 (0.01)	0.64 (0.00)	0.03 (0.01)	0.31 (0.00)	0.04 (0.01)	0.34 (0.01)
Task-relatedness	0.34 (0.01)	0.67 (0.00)	0.04 (0.01)	0.20 (0.00)	0.05 (0.01)	0.22 (0.01)
Freely moving	0.21 (0.01)	0.61 (0.01)	0.02 (0.00)	0.25 (0.00)	0.04 (0.01)	0.29 (0.01)
Goal-oriented	0.32 (0.01)	0.66 (0.00)	0.03 (0.01)	0.19 (0.00)	0.12 (0.01)	0.27 (0.01)
Sticky	0.28 (0.01)	0.64 (0.01)	0.05 (0.01)	0.32 (0.00)	0.09 (0.01)	0.36 (0.01)
Self-oriented	0.23 (0.01)	0.59 (0.01)	0.01 (0.01)	0.20 (0.00)	0.04 (0.01)	0.24 (0.01)
Others-oriented	0.38 (0.01)	0.69 (0.00)	0.01 (0.01)	0.23 (0.00)	0.03 (0.01)	0.27 (0.01)
Visual modality	0.21 (0.01)	0.60 (0.00)	0.01 (0.01)	0.32 (0.00)	0.08 (0.01)	0.33 (0.01)
Auditory modality	0.27 (0.01)	0.64 (0.01)	0.00 (0.01)	0.22 (0.00)	0.05 (0.01)	0.27 (0.01)

MCC: Matthew’s correlation coefficient; BA: balanced accuracy. The classification performance as indexed by two performance metrics is reported for the idiographic and nomothetic groups, representing the detection of a given thought dimension based on task-at-hand. Each value represents the mean (and standard deviation) classification performance across the 100 iterations. The first two sets of columns show the classification performance based on the original data from idiographic group and nomothetic group, and the third set of columns show the classification performance based on up-sampled data in nomothetic group (such that the number of data points approximates that in the idiographic group). Classification performance for all thought dimensions in the idiographic group was significantly better than the nomothetic group (original data at p <.001 and up-sampled data at p <.05)

In addition to the group-level results, we also report the individual-level classification performance for each participant in the idiographic group in Supplementary Table S18. There is a wide range among the seven participants; for instance, the MCC for off-task thoughts ranged from 0.07 to 0.75. A similar pattern was observed for all thought dimensions, such that classification performance was at chance level for one to two participants and at similar or superior levels as the group mean for the remaining participants. In line with our findings with the primary analyses, these results highlight the idiosyncratic nature of the modulatory effects of task on different dimensions of thoughts, suggesting that in general it is possible to detect an individual’s thoughts based on their ongoing task but there is considerable individual variability in their levels of classification performance.

Finally, having established that the precision experiences sampling approach enhanced person-specific predictions, we then verified the generalizability of the model in the idiographic group by examining whether it was possible to predict ongoing thoughts based on group-level data (across-participant model). Overall, the across-participant models showed chance-level classification performance for some thought dimensions, including internally oriented, freely moving, goal-oriented, visual and auditory thoughts (MCC, *M*s = 0.00 to 0.07). The remaining thought dimensions, including task-relatedness, sticky, self-oriented, others-oriented, showed decent classification performance (MCC, *M*s = 0.23 to 0.33). Notably, within-participant models showed better classification performance on all thought dimensions compared to the across-participant models, with the exception for self-oriented thoughts in which the opposite pattern was observed. These results show that the model is generalizable for only a subset of thought dimensions. They also indicate that the precision experience sampling idiographic approach led to enhanced accuracy when making predictions about ongoing thoughts based on the individual’s own data but less so for group-level data, suggesting that the precision experience sampling approach is generally more beneficial for person-specific than across-participant predictions. The results of these analyses are reported in Supplementary Table S19.

## Discussion

In the current study, we first compared ongoing thoughts between the idiographic approach and the nomothetic approach, in order to validate the idiographic dataset. We then examined individual differences in the modulatory effects of task on ongoing thoughts and whether these individual patterns correspond to group-level patterns in the idiographic group, as well as the utility of the idiographic approach with precision experience sampling in enhancing person-specific predictions of ongoing thoughts based on the task-at-hand using machine learning. We found that participants in the idiographic group exhibited different patterns of task-modulated ongoing thoughts, such that the specific tasks that impacted a given thought dimension differed across participants, suggesting considerable individual differences in task-modulatory effects on ongoing thought. These individual-level patterns in the idiographic group, however, did not correspond well with those observed at the group level in many cases. Furthermore, acquiring more data from an individual in the idiographic group improved the classification of that individual’s ongoing thoughts based on the task-at-hand using machine learning compared to the nomothetic group, underscoring the benefits of a idiographic approach (Beltz et al., 2016) with precision experience sampling. We discuss the implications below.

### Task-at-hand differentially predicts ongoing thoughts across individuals

The modulatory effects of task on ongoing thoughts were observed at the individual level as well as at the group level in the idiographic group. At the individual level, some thought dimensions showed more consistent task-modulatory effects across individuals than others. As an example, there was a significant effect of task for at least half of the participants for goal-oriented thoughts, off-task thoughts, others-oriented thoughts and visual thoughts. These results are in line with past literature reporting the influence of contextual factors on the prevalence of off-task thoughts (Smallwood & Andrews-Hanna, 2013; Turnbull et al., 2019) and visual thoughts (Choi et al., 2017) at the group level. In contrast, only one participant showed a significant effect of task on sticky thought. These patterns suggest that some thought dimensions are more robustly modulated by the task-at-hand, whereas other thought dimensions are less impacted by the task and may instead be impacted by other factors. Our results are largely consistent with existing literature demonstrating that our thought patterns change based on the task being performed (Chitiz et al., 2023; Mckeown et al., 2025; Mulholland et al., 2024) and further demonstrate there are considerable individual differences in these task-modulatory effects of thoughts.

Critically, there were notable differences in how specific tasks modulated ongoing thoughts across individuals, highlighting that although the modulatory effects of task on ongoing thoughts were observed across participants at the individual level, the manner in which a task modulates their thoughts differed. These individual-level analyses revealed not only that the same task elicited different types of thoughts within individuals but also that the patterns observed at the group level did not always accurately characterize the individual patterns (such that it does for some individuals but not for others). For instance, playing games was associated with the highest level of off-task thought numerically at the group level, but it predicted relatively lower levels of off-task thought in three participants compared to other tasks. As another example, rewatching videos was associated with the lowest levels of off-task thoughts at the group level, but this was only observed across one participant at the individual level. This notion of distinct individual profiles of ongoing thoughts aligns with reports of unique patterns of classification accuracy and functional connectivity of neural regions within individuals that did not generalize to others (Hung & Hsieh, 2022; Kucyi et al., 2024). These results collectively highlight the idiosyncratic characteristics of our thoughts as well as how situational factors impact them. They further underscore the importance of considering the uniqueness of each individual’s thoughts in applied settings (such as education or mental health interventions) *in addition* to the general group-level patterns that are used for theory building and describing the general population (Shareef—Trudeau et al., 2025).

Taken together, although the task-at-hand appears to consistently modulate the types of thoughts we engage in at the individual level, our results indicate that these task-related modulations operate differently across individuals and that these individual patterns do not always correspond to group-level patterns. These findings corroborate existing literature that emphasizes the importance of examining and considering individual differences (Cheung et al., 2017, p. 201; Molenaar, 2004; Welhaf et al., 2020), especially how they interact with situational factors (Beck & Jackson, 2022). They highlight the complementary nature of the idiographic and nomothetic approaches, as the nomothetic approach reveals the patterns that are common across individuals, and the idiographic approach reveals patterns that are specific to a given individual. Importantly, that the results of these approaches do not always correspond cautions against the assumption that the relationships between tasks and ongoing thoughts derived from aggregate data generalize well to any given individual.

### Superior classification performance of ongoing thoughts within individuals

In acquiring more data points for each participant, the precision experience sampling idiographic approach enhanced classification performance for ongoing thoughts based on the task-at-hand. This was observed when making person-specific predictions using the k-fold within-subject strategy in the idiographic group compared to the nomothetic group. Overall, the others-oriented thought dimension showed the best performance, followed by off-task thoughts and goal-oriented thoughts in the idiographic group. Similar to the above analyses, we also found considerable individual variability in the classification performance across each of the seven participants, which further highlights the idiosyncratic nature of the relationship between ongoing task and dimensions of thoughts. Notably, our overall classification performance for off-task thoughts, which is the most commonly examined thought dimension using machine learning, was at least comparable and at times superior to existing literature that used physiological measures as features (Dong et al., 2021; Jin et al., 2019; Kam et al., 2025; Rahnuma et al., 2024). Given that the idiographic group had more data points per participant compared to the nomothetic group, these results suggest that we can predict with higher accuracy an individuals’ thoughts based on their task with a larger number of data points from that individual. It is important, however, to point out the individual variability in the classification performance across participants in the idiographic group, suggesting that the increased accuracy that accompanies more data may not necessarily be observed for every thought dimension in a given individual. Notably, we observed higher accuracy in the idiographic group in comparison with the original data as well as after data augmentation in the nomothetic group, suggesting that the difference in performance may not be solely attributed to the reduced number of data points per se in the nomothetic group. It is possible that the up-sampling of data points from a small pool of data per participant introduced noise as well as stability, which did not improve classification performance in the nomothetic group.

The enhanced classification performance was also observed when comparing the within-participant approach to the across-participant approach in the idiographic group. Specifically, we found that having additional data points per participant mainly benefited the classification of thoughts for that participant, and to a lesser extent when using others’ data to predict a given participant’s thoughts. This suggests that the precision experience sampling approach selectively improved the accuracy of person-specific predictions with less impact on across-participant predictions.

Collectively, this suggests that increasing data points in participants based on real data is critical for maximizing classification accuracy within participants. In other words, obtaining a large number of data points across time and context for a given individual enhanced the prediction of that individual’s ongoing thoughts as a function of the task-at-hand. There is, however, considerable individual variability in classification performance across participants and thought dimensions, suggesting that this benefit in classification performance from increasing data points may not apply to all individuals and all thought dimensions. Although our results are in line with the common conception that person-specific predictions are generally more effective than group-level predictions, one notable point of consideration is the relatively small amount of data obtained for each participant in the idiographic group. In other words, the better classification performance observed in the idiographic group was gained through a small number of datasets (n = 7) per participant. This highlights the value of the precision experience sampling idiographic approach in improving the accuracy of person-specific predictions of one’s thoughts.

### Ongoing thought measures were not consistently more reliable in the idiographic group

The reliability of the reports of ongoing thoughts is higher in the idiographic group for some thought dimensions and higher in the nomothetic group for others. These findings only partially corroborate existing studies reporting higher reliability with the idiographic approach (Beltz et al., 2016), indicating that this benefit of the idiographic approach only applies to certain types of thoughts. Our data suggest that the usual benefits of increased reliability with increased data points may be offset by the naturally fluctuating qualities of some of the dimensions of ongoing thoughts. In other words, if the dimensions of thoughts being measured are not consistent over time, then increasing data points does not necessarily increase the reliability of the assessment. Interestingly, the nature of the thought dimensions shed some light on why some may be more reliable over time than others.

The thought dimensions that showed higher reliability in the idiographic group (i.e., internally oriented, off-task, freely moving, goal-oriented, self- and others-oriented) tend to be related to the dynamics or content of thoughts, indicating that what we think about (i.e., content) and how thoughts unfold over time (i.e., dynamics) are more stable within individuals. In contrast, the thought dimensions that are comparable or showed higher reliability in the nomothetic group (i.e., sticky, visual and auditory thought dimensions) tend to be more saliency or sensory driven, indicating that what we pay attention to and how stuck we are on certain thoughts (i.e., saliency driven) and how we think in terms of form (i.e., sensory driven) are less consistent within individuals. This suggests that the content and dynamics of our thoughts are more reliable with more data acquired across multiple sessions in participants, whereas thoughts that are more salient or sensory driven appear to require less data to be more reliable.

Thoughts that were saliency driven (e.g., sticky thoughts) or sensory driven (e.g., visual versus auditory modality) may be more variable because they are likely to depend more on external, situational factors. For instance, individuals would be more likely to experience sticky thoughts if something personally salient happened in life, causing their thoughts to get stuck on a certain topic (e.g., getting into an argument with a partner or family member), but may otherwise not have sticky thoughts. Studies have also shown that the modality of the task modulates the modality of our thoughts (Choi et al., 2017) and the modality of our thoughts are not stable over time as assessed across two sessions (Öncel et al., 2024), suggesting that whether we think in images or words depends in part on our task and may therefore explain why these thoughts dimensions may be less consistent in the idiographic participants if they complete different tasks across the multiple sessions. Accordingly, another potential explanation is that the reliability assessment does not take into account the impact of contextual or situational factors, such as the current task or personally salient events in life. In particular, given that the task-at-hand modulates the types of thoughts we engage in, it is possible that the reliability measure depends on the extent to which a given thought dimension is strongly modulated by the task-at-hand and the frequency with which the task was being performed across individuals in each study. This suggests the measure of reliability of thought dimensions may be sensitive to task- or context-dependent effects, highlighting the importance of accounting for task or context broadly in implementing these measures. Future investigations that involve a pre-determined set of experimental tasks may more optimally address the reliability of thought dimensions within specific contexts. This approach has been recently implemented in a study that used a pre-determined set of 14 highly controlled cognitive tasks to systematically map psychological states across task contexts (Mckeown et al., 2025). Convergence between controlled experimental paradigms along with the naturalistic paradigms employed in this study would further strengthen these patterns of findings concerning the contextualization of ongoing thoughts and experiences.

### Similar thought patterns across the idiographic and nomothetic groups

Finally, the overall patterns of ongoing thoughts were generally similar across the idiographic and nomothetic groups, though some differences emerged. Specifically, the relative levels of thought dimension ratings appeared to be comparable between groups, despite differences in the absolute levels of thought dimensions at the group level. Moreover, the correlation matrices were also significantly similar between groups. Importantly, the task-modulatory effects on thought dimensions were similarly observed in both groups. These results indicate that the relative levels of ongoing thoughts, the relationship between thought dimensions, as well as the effects of task on thoughts observed in the smaller sample in the idiographic group were similar to patterns observed in the larger sample in the nomothetic group, which provides some support for the generalizability of the data from the idiographic group. Although we observed differences in the absolute values of several thought dimension ratings between groups, these differences did not correspond to the robustness of task-related effects of thought dimensions across individuals in the idiographic group; therefore, the effects observed in ongoing thoughts at the individual level are unlikely attributable to differences in the levels of thought dimensions between groups.

### Limitations and future directions

In interpreting our findings, one consideration concerns the specific thought dimensions we queried in our study. Although we inquired about a wide range of thought dimensions consistent with past studies (Mckeown et al., 2021; Mills et al., 2018; Mulholland et al., 2023; Wang et al., 2018), they are not exhaustive and may not fully characterize the ongoing thoughts of individuals, especially those with clinical or neurological conditions. Our results may therefore not generalize beyond the thought dimensions we examined. A direction for future research would be to further explore additional thought dimensions in the general population as well as clinically relevant thought dimensions in clinical populations. Relatedly, as the current study is exploratory in nature, we did not strictly control for all sources of family-wise error rate inflation in our main analyses; future confirmatory research with a larger sample and more conservative corrections would be warranted. Such a study could explore more sophisticated analytical approaches to account for multiple thought dimensions as dependent variables, such as multivariate or hierarchical linear mixed effects models, as well as reducing the number of thought dimensions considered, either by using results here to select dimensions of interest or by designing thought probe questions with dimension reduction techniques in mind (e.g., Chitiz et al., 2025; Mckeown et al., 2025).

A second point of consideration is that different individuals were recruited for the idiographic and nomothetic groups. Therefore, in the preliminary analyses where we compared the two groups, we were also comparing different individuals. Although the core findings involving individual-level analyses within the idiographic group would not be impacted by differences across groups, the possibility remains that the differences in the absolute levels of thought dimension ratings observed across groups may be attributable to individual differences across groups rather than methodological approaches. However, we highlight that the relative levels of ongoing thoughts as well as the relationships between thought dimensions were comparable between studies. An important avenue for future research involves recruiting the same individuals for assessing both the idiographic and nomothetic approaches to address this (Beltz et al., 2016; Fisher et al., 2017). Notably, this comparison between idiographic and nomothetic groups was particularly relevant for our study with a smaller sample in the idiographic group but would be less of a concern in future studies implementing the precision experience sampling idiographic approach on a sufficiently large sample.

A related consideration concerns the sample size and diversity of both groups. Although we have multiple sessions of data from each participant and our sample size is comparable to studies with similar objectives (Hung & Hsieh, 2022; Kucyi et al., 2024), our sample size in the idiographic group is small (n = 7). To address this, we verified the patterns of ongoing thoughts and the task-modulatory effects on thoughts in this group by showing that they are comparable to a larger sample in the nomothetic group (n = 49), though the sample size of 49 may still be considered as relatively small. Another consideration is that both samples were recruited in a Western, Educated, Industrialized, Rich, and Democratic (W.E.I.R.D.; Henrich et al., 2010) society, thereby limiting the generalization of our results. Future investigations should aim to replicate these findings in a larger, and more diverse, sample.

Finally, our study aimed to examine modulatory effects of task on ongoing thoughts in a naturalistic setting, prioritizing ecological validity over experimental constraints. With our approach, experimenters have minimal control over the nature of the tasks, the duration with which a given task was performed and the number of tasks engaged during each session. These are all important task variables that may be relevant to the question at hand and may account for additional variance in our models. Future studies that use a pre-determined set of experimentally controlled cognitive tasks to map ongoing thoughts across task contexts (similar to Mckeown et al., 2025) may be optimally suited to parameterize these variables.

## Conclusions

In summary, our experimental design involving multiple recording sessions for each participant afforded us the opportunity to examine patterns of ongoing thoughts at the individual level, highlighting the value of the precision experience sampling idiographic approach. Our findings revealed substantial individual differences in the task-modulatory effects on ongoing thoughts, and that the patterns observed at the group level did not always accurately represent individual-level patterns. This highlights the importance of considering both individual and contextual factors (such as the task-at-hand) in making person-specific predictions about future thoughts. Notably, obtaining a large amount of data from an individual improved our ability to predict that individual’s ongoing thoughts based on the task-at-hand using machine learning. The capacity to enhance the accuracy in making person-specific predictions about one’s thoughts using the precision experience sampling idiographic approach has important clinical and real-world applications. Knowing when an individual engages in certain thought patterns under which contexts can inform personalized strategies that aim to increase cognitive and affective functions on an individual basis. In turn, these person-specific prevention and intervention programs have the potential to improve learning outcomes in students in educational settings and enhance treatment outcomes in patients in medical and clinical contexts, exemplifying the wide range of implications of precision experience sampling.

## Supplementary Information

Supplementary Material 1.

## Data Availability

The data that support the findings of this study as well as the code relevant to this study are available at https://osf.io/7a8tv/?view_only=fdcfece02c9f40d2946d3e355fbf32bf. This study was not pre-registered.
