# Supplementary material for: Capturing naturalistic thoughts using a precision experience sampling idiographic approach
Source: Cogn Res Princ Implic. 2026 Apr 15;11:33. doi: 10.1186/s41235-026-00728-8 (PMC13083714; doi:10.1186/s41235-026-00728-8)
Supplement: Supplementary file 1 — Supplementary Material 1. [file 41235_2026_728_MOESM1_ESM.docx]

**Capturing Naturalistic Thoughts using a**

**Precision Experience Sampling Idiographic Approach**

**Supplementary Materials**

Julia W. Y. Kam, Sairamya Nanjappan Jothiraj, Emily Beauchemin, Nabil Al Nahin Ch, Laura C. Allen, Jolie B. Wormwood, Caitlin Mills

**Supplementary Results**

**Descriptives of Tasks**

In the idiographic group, participants reported on average spending more time on some tasks than others (reading or studying: 33%, video watching: 18%, writing: 11%, web surfing: 8%, playing games: 13%, and other cognitively demanding tasks: 16%). The nomothetic group reported a similar pattern (reading or studying: 37%, video watching: 24%, writing: 12%, web surfing: 10%, playing games: 4% and other cognitively demanding tasks: 14%).

**Supplementary Figures**

**Supplementary Figure S1**

*Individual and Group Level Ratings of Each Thought Dimension*

*Note:* The mean ratings (along with the standard error of the mean) were computed across participants in the idiographic group and nomothetic group and across sessions within participants for each of the seven participants in the idiographic group. The ratings ranged from 1 = not at all to 7 = extremely for all thought dimensions except for internally-oriented thoughts (1 = internally oriented to 7 = externally oriented) and off-task thoughts (1 = on-task to 7 = off-task). Int = internally-oriented; off = off-task; vis = visual; aud = auditory.**Supplementary Figure S2**

*Probe-Level Ratings of Thought Dimensions for all Sessions for Exemplar Participant*

**
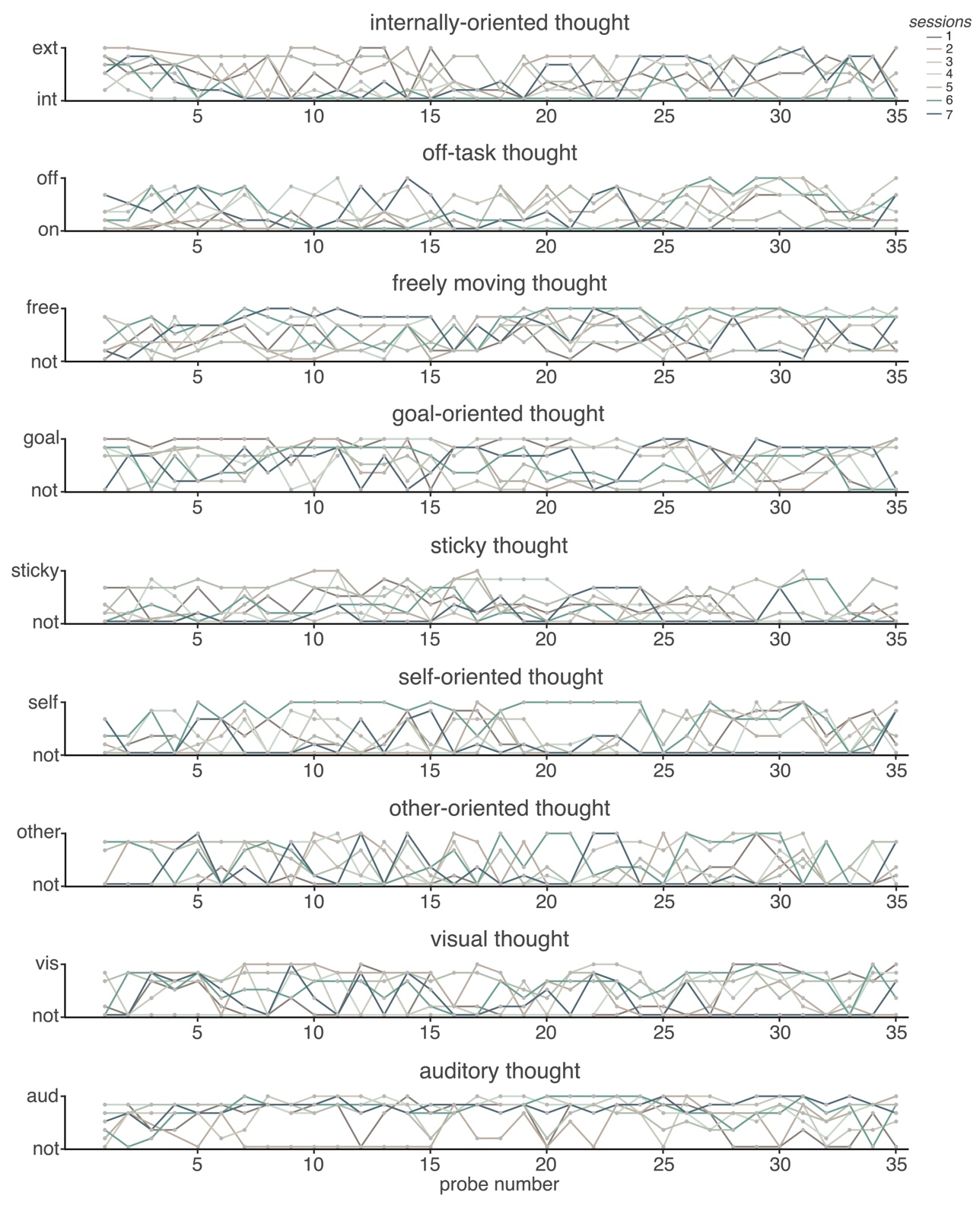
**

*Note:* The ratings on a 7-point Likert scale are shown for each thought dimension for each probe within each of the seven sessions for Participant 1 in the idiographic group.

**Supplementary Tables**

**Supplementary Table S1**

*Definitions and example scenarios corresponding to each question asked during the multi-dimensional experience sampling thought probes.*

| **Thought Dimension** | **Description** |
| --- | --- |
| What is your current task? | “Task” refers to whatever it is you’re supposed to be doing. This could be actual work or doing a google search or watching a video. This doesn’t need to be a physical or “productive” task (e.g., if you’re intending to daydream, then your task is daydreaming).  This question prompts you to type in a response, which can be a brief description consisting of a few words. |
| To what extent were your thoughts focused on your inner thoughts or external stimuli? | Internal implies that your thoughts were internally focused, for example if you were doing a math problem in your head or recalling memories of past vacations   - you might respond **1 = mostly inner** **thoughts**, if your thoughts were strictly focused on one topic (e.g., the pandemic or career plans).   External implies that your thoughts were focused on an external stimulus, either visual, auditory or tactile like the image you’re seeing on the screen, or the sensation of the EEG cap on your head.   - you might respond **7 = mostly external stimuli,** if your thoughts were exclusively focused on an external stimulus over any internal thoughts |
| To what extent were your thoughts on-task versus off-task? | Based on the task you listed in the previous question, were you paying attention to the task or not.  Scale: completely on-task to off-task  Examples:   - you might respond **1 = Very much on-task**, if you were completely focused on finishing your assigned task for class (your task). - you might respond **7 = Very much off-task,**if your thoughts were not focused on reading your e-book (your task) at all, and instead focused on planning your next vacation |
| To what extent were your thoughts freely moving? | Freely moving means your thoughts wandered through different topics with no overarching purpose or direction, and thoughts were not focused on anything for too long.  Examples:   - you might respond **1 = not at all**, if your thoughts were strictly focused on one topic (e.g., the pandemic or career plans). - you might respond **7 = all the time,**if your thoughts wandered swiftly from your future plans to the concept of equality to childhood memories to your friend’s wedding and did so for the entire 5 minutes. |
| To what extent were your thoughts difficult to disengage from? | An inability to disengage means that you can’t help but think about that topic, and can’t stop if you want to; your thoughts are drawn to this topic, whether or not you’re actively trying to think about it  Examples:   - you might respond **1 = not at all**, if you were not stuck on any topic and were able to easily stop thinking about it and move on to another thought. - you might respond **7 = all the time,**if you couldn’t help but think about your sibling who got into a car accident. No matter how hard you tried your thoughts were stuck on this topic for the entire 5 minutes. |
| To what extent were your thoughts goal-directed on a topic? | Goal-directed means that your thoughts are focused on an overarching goal, and that when you have thoughts that distract you from your goal, you bring your mind back on track  Examples:   - you might respond **1 = not at all**, if you were watching a video or browsing the internet with no particular goal, and your attention to these activities wanders aimlessly. - you might respond **7 = all the time,**if you were very focused on imagining the sequence of movements in your dance routine as rehearsal for your upcoming dance recital. |
| To what extent were your thoughts about yourself? | Examples:   - you might respond **1 = not at all**, if you were only thinking about your sibling and their dog and not thinking about yourself at all. - you might respond **7 = all the time,** if your thoughts were focused on yourself, like what you want to do in a future career, and not related to someone else. |
| To what extent were your thoughts about another person/other people? | Examples:   - you might respond **1 = not at all**, if your thoughts were focused on yourself, or an abstract topic like art or university policy, and not related to someone else. - you might respond **7 = all the time,** if you were thinking about someone else, like your friend or parents, and not yourself during the task. |
| To what extent were your thoughts in the visual modality (whether it be external or internal)? | Examples:   - you might respond **1 = not at all**, if you weren’t seeing the cross at all anymore, but were completely focused on a song in your head. - you might respond **7 = all the time,**if you were thinking about all the details of that tiny cross, and trying to measure it in your mind |
| To what extent were your thoughts in the auditory modality (whether it be external or internal)? | Examples:   - you might respond **1 = not at all**, if you were completely focused on the cross and found that you zoned out the sound of the experimenter nearby. - you might respond **7 = all the time,**if you were staring blankly while replaying a speech you had heard in your head |

**Supplementary Table S2**

*Comparisons of Mean and Standard Deviation of Thought Dimension Ratings between the Idiographic Group and Nomothetic Group*

| **Thought** | **Mean Descriptives** | | **Group** | |
| --- | --- | --- | --- | --- |
| **Dimension** | ***Idiographic*** | ***Nomothetic*** | $\boldsymbol{b}$ **(SE)** | $\boldsymbol{\chi}$**^2^** |
| Mean Ratings |  |  |  |  |
| Internal-External | 3.90 | 4.19 | 0.28 (0.19) | 2.21 |
| Task-relatedness | 3.04 | 1.63 | -1.41 (0.16) | 74.11** |
| Freely moving | 3.63 | 2.16 | -1.47 (0.16) | 83.82** |
| Goal-oriented | 5.81 | 4.08 | -1.73 (0.19) | 81.02** |
| Sticky | 2.45 | 1.75 | -0.70 (0.15) | 21.30** |
| Self-oriented | 3.31 | 1.84 | -1.47 (0.17) | 71.18** |
| Others-oriented | 2.39 | 3.35 | 0.96 (0.20) | 23.60** |
| Visual modality | 3.47 | 3.78 | 0.31 (0.19) | 2.72 |
| Auditory modality | 6.29 | 4.48 | -1.81 (0.17) | 109.73** |
| SD Ratings |  |  |  |  |
| Internal-External | 1.42 | 1.45 | 0.03 (0.17) | 0.08 |
| Task-relatedness | 1.41 | 1.29 | -0.12 (0.18) | 0.46 |
| Freely moving | 1.36 | 1.31 | -0.05 (0.14) | 0.08 |
| Goal-oriented | 1.44 | 1.55 | 0.11 (0.20) | 0.38 |
| Sticky | 1.33 | 0.94 | -0.40 (0.18) | 4.99* |
| Self-oriented | 1.26 | 1.43 | 0.16 (0.20) | 0.67 |
| Others-oriented | 1.44 | 1.62 | 0.18 (0.23) | 0.65 |
| Visual modality | 1.40 | 1.49 | 0.09 (0.20) | 0.22 |
| Auditory modality | 1.23 | 1.45 | 0.23 (0.16) | 2.24 |

*Note: Mean descriptives reflect marginal means from the models.* $\chi$^2^ *represents the main effect of “group” in each column based on likelihood ratio tests, ** p < .001, * p < .05. This table reports the main effect of study controlling for age in separate models for mean ratings (top) and the standard deviation (SD) of ratings (bottom).*

**Supplementary Table S3**

*Comparisons of Mean and Standard Deviation of Thought Dimension Ratings between the Idiographic Group and Nomothetic Group Accounting for Number of Tasks*

| **Thought** | **Mean Descriptives** | | **Group** | |
| --- | --- | --- | --- | --- |
| **Dimension** | ***Idiographic*** | ***Nomothetic*** | $\boldsymbol{b}$ **(SE)** | $\boldsymbol{\chi}$**^2^** |
| Mean Ratings |  |  |  |  |
| Internal-External | 3.90 | 4.18 | 0.28 (0.19) | 2.17 |
| Task-relatedness | 3.05 | 1.63 | -1.42 (0.16) | 74.45** |
| Freely moving | 3.63 | 2.16 | -1.47 (0.16) | 83.81** |
| Goal-oriented | 5.81 | 4.09 | -1.73 (0.19) | 81.11** |
| Sticky | 2.45 | 1.75 | -0.70 (0.15) | 21.26** |
| Self-oriented | 3.31 | 1.84 | -1.46 (0.17) | 70.94** |
| Others-oriented | 2.39 | 3.35 | 0.96 (0.20) | 23.73** |
| Visual modality | 3.47 | 3.78 | 0.31 (0.19) | 2.71 |
| Auditory modality | 6.29 | 4.48 | -1.81 (0.17) | 110.14** |
| SD Ratings |  |  |  |  |
| Internal-External | 1.44 | 1.44 | -0.01 (0.18) | 0.004 |
| Task-relatedness | 1.41 | 1.29 | -0.12 (0.18) | 0.46 |
| Freely moving | 1.36 | 1.31 | -0.05 (0.15) | 0.10 |
| Goal-oriented | 1.44 | 1.55 | 0.11 (0.20) | 0.38 |
| Sticky | 1.33 | 0.94 | -0.40 (0.18) | 5.30* |
| Self-oriented | 1.27 | 1.43 | 0.16 (0.20) | 0.69 |
| Others-oriented | 1.45 | 1.62 | 0.17 (0.22) | 0.64 |
| Visual modality | 1.41 | 1.49 | 0.08 (0.20) | 0.21 |
| Auditory modality | 1.25 | 1.46 | 0.21 (0.17) | 1.62 |

*Note: Mean descriptives reflect marginal means from the models.* $\chi$^2^ *represents the main effect of “group” in each column based on likelihood ratio tests, ** p < .001, * p < .05. This table reports the main effect of study controlling for age and number of tasks in separate models for mean ratings (top) and the standard deviation (SD) of ratings (bottom).*

**Supplementary Table S4**

*Comparisons of Mean and Standard Deviation of Thought Dimension Ratings between the Idiographic Group (during Session 1) and Nomothetic Group*

| **Thought** | **Mean Descriptives** | | **Group** | |
| --- | --- | --- | --- | --- |
| **Dimension** | ***Idiographic*** | ***Nomothetic*** | $\boldsymbol{b}$ **(SE)** | $\boldsymbol{\chi}$**^2^** |
| Mean Ratings |  |  |  |  |
| Internal-External | 4.43 | 3.57 | -0.85 (0.26) | 10.72** |
| Task-relatedness | 3.07 | 2.19 | -0.88 (0.22) | 15.78** |
| Freely moving | 3.57 | 2.68 | -0.90 (0.22) | 16.71** |
| Goal-oriented | 6.82 | 4.54 | -2.29 (0.26) | 75.79** |
| Sticky | 2.88 | 2.17 | -0.71 (0.20) | 12.91** |
| Self-oriented | 3.00 | 2.42 | -0.58 (0.24) | 5.92* |
| Others-oriented | 1.50 | 3.12 | 1.61 (0.25) | 40.38** |
| Visual modality | 3.27 | 3.60 | 0.33 (0.25) | 1.70 |
| Auditory modality | 4.71 | 4.27 | -0.44 (0.24) | 3.28 |
| SD Ratings |  |  |  |  |
| Internal-External | 1.51 | 1.55 | 0.05 (0.30) | 0.01 |
| Task-relatedness | 1.52 | 1.46 | -0.06 (0.29) | 0.04 |
| Freely moving | 1.38 | 1.45 | 0.06 (0.25) | 0.07 |
| Goal-oriented | 1.58 | 1.65 | 0.08 (0.30) | 0.08 |
| Sticky | 1.52 | 1.16 | -0.36 (0.29) | 1.59 |
| Self-oriented | 1.30 | 1.48 | 0.18 (0.34) | 0.31 |
| Others-oriented | 1.09 | 1.71 | 0.62 (0.33) | 3.40 |
| Visual modality | 1.60 | 1.57 | -0.04 (0.31) | 0.02 |
| Auditory modality | 1.69 | 1.51 | -0.18 (0.29) | 0.38 |

*Note: Mean descriptives reflect marginal means from the models.* $\chi$^2^ *represents the main effect of “group” in each column based on likelihood ratio tests, ** p < .001, * p < .05. This table reports the main effect of study controlling for age, including only session 1 from the idiographic group, in separate models for mean ratings (top) and the standard deviation (SD) of ratings (bottom). Although this analysis serves to compare participants from both groups during their first session (which is also the only session for the nomothetic group), we caution readers that based on substantial differences in sample size, we cannot confidently conclude from this analysis whether or not the originally reported similarities/differences in the variability of thought dimension ratings between groups in Supplementary Table S2 were due to effects of multiple sessions in the idiographic group.*

**Supplementary Table S5**

*Proportion of Tasks Engaged by Participant across Sessions in the Idiographic Group and across Participants in the Nomothetic Group*

| **Participants** | **Read** | **Video** | **Write** | **Web** | **Game** | **Others** |
| --- | --- | --- | --- | --- | --- | --- |
| Participant 1 | 0.56 | 0.21 | 0.17 | 0.05 | 0 | 0 |
| Session 1 | 0.31 | 0.20 | 0.40 | 0.09 | 0 | 0 |
| Session 2 | 0.39 | 0.48 | 0 | 0.12 | 0 | 0 |
| Session 3 | 0.94 | 0 | 0 | 0.06 | 0 | 0 |
| Session 4 | 0.69 | 0 | 0.31 | 0 | 0 | 0 |
| Session 5 | 0.14 | 0.83 | 0 | 0.03 | 0 | 0 |
| Session 6 | 0.46 | 0 | 0.49 | 0.06 | 0 | 0 |
| Session 7 | 1 | 0 | 0 | 0 | 0 | 0 |
| Participant 2 | 0.32 | 0.57 | 0.05 | 0.06 | 0 | 0 |
| Session 1 | 0.83 | 0 | 0 | 0.17 | 0 | 0 |
| Session 2 | 0 | 1 | 0 | 0 | 0 | 0 |
| Session 3 | 0.77 | 0 | 0 | 0.23 | 0 | 0 |
| Session 4 | 0.20 | 0.80 | 0 | 0 | 0 | 0 |
| Session 5 | 0.41 | 0.21 | 0.38 | 0 | 0 | 0 |
| Session 6 | 0 | 1 | 0 | 0 | 0 | 0 |
| Session 7 | 0 | 1 | 0 | 0 | 0 | 0 |
| Participant 3 | 0.14 | 0 | 0.14 | 0.13 | 0.46 | 0.13 |
| Session 1 | 0.16 | 0 | 0.25 | 0 | 0 | 0.59 |
| Session 2 | 0 | 0 | 0 | 0.03 | 0.69 | 0.29 |
| Session 3 | 0.26 | 0 | 0.20 | 0.31 | 0.23 | 0 |
| Session 4 | 0.15 | 0 | 0.35 | 0.26 | 0.24 | 0 |
| Session 5 | 0.20 | 0 | 0 | 0.11 | 0.69 | 0 |
| Session 6 | 0.21 | 0 | 0.21 | 0.15 | 0.41 | 0.03 |
| Session 7 | 0.03 | 0 | 0 | 0.03 | 0.94 | 0 |
| Participant 4 | 0.17 | 0.04 | 0.34 | 0.15 | 0.01 | 0.29 |
| Session 1 | 0.06 | 0 | 0.80 | 0.03 | 0 | 0.11 |
| Session 2 | 0.23 | 0 | 0.03 | 0.14 | 0.06 | 0.54 |
| Session 3 | 0.14 | 0.29 | 0.04 | 0.46 | 0 | 0.07 |
| Session 4 | 0 | 0.03 | 0.79 | 0.18 | 0 | 0 |
| Session 5 | 0.36 | 0 | 0.12 | 0.06 | 0 | 0.45 |
| Session 6 | 0.03 | 0 | 0.35 | 0.06 | 0 | 0.56 |
| Session 7 | 0.34 | 0 | 0.20 | 0.17 | 0.03 | 0.26 |
| Participant 5 | 0.19 | 0.19 | 0.06 | 0.11 | 0.44 | 0.004 |
| Session 1 | 0.21 | 0.79 | 0 | 0 | 0 | 0 |
| Session 2 | 0.06 | 0.50 | 0 | 0.03 | 0.41 | 0 |
| Session 3 | 0.20 | 0 | 0 | 0.06 | 0.74 | 0 |
| Session 4 | 0.07 | 0 | 0 | 0.59 | 0.31 | 0.03 |
| Session 5 | 0.54 | 0 | 0.40 | 0.06 | 0 | 0 |
| Session 6 | 0 | 0 | 0 | 0.09 | 0.91 | 0 |
| Session 7 | 0.26 | 0 | 0 | 0.06 | 0.69 | 0 |
| Participant 6 | 0.65 | 0.26 | 0 | 0.06 | 0.004 | 0.02 |
| Session 1 | 0.14 | 0.80 | 0 | 0.06 | 0 | 0 |
| Session 2 | 0 | 1 | 0 | 0 | 0 | 0 |
| Session 3 | 1 | 0 | 0 | 0 | 0 | 0 |
| Session 4 | 0.85 | 0 | 0 | 0.12 | 0 | 0.03 |
| Session 5 | 0.69 | 0 | 0 | 0.17 | 0.03 | 0.1 |
| Session 6 | 0.89 | 0 | 0 | 0.09 | 0 | 0.03 |
| Session 7 | 1 | 0 | 0 | 0 | 0 | 0 |
| Participant 7 | 0.30 | 0 | 0.04 | 0.004 | 0.004 | 0.66 |
| Session 1 | 0.03 | 0 | 0 | 0.03 | 0 | 0.94 |
| Session 2 | 0.39 | 0 | 0.30 | 0 | 0.03 | 0.27 |
| Session 3 | 0.31 | 0 | 0 | 0 | 0 | 0.69 |
| Session 4 | 0 | 0 | 0 | 0 | 0 | 1 |
| Session 5 | 0 | 0 | 0 | 0 | 0 | 1 |
| Session 6 | 0.31 | 0 | 0 | 0 | 0 | 0.69 |
| Session 7 | 1 | 0 | 0 | 0 | 0 | 0 |
| Idiographic Group Avg | 0.33 | 0.18 | 0.11 | 0.08 | 0.13 | 0.16 |
| Nomothetic Group Avg | 0.37 | 0.24 | 0.12 | 0.10 | 0.04 | 0.14 |

*Note: The means of the proportions for individual participants are reported across all seven sessions whereas the means of the proportions for groups are reported across all participants within that group. The total proportion does not necessarily add up to 1 because miscellaneous activities and missing responses were not included.*

**Supplementary Table S6**

*Reliability of Thought Dimension Ratings in Idiographic and Nomothetic Groups indexed by Intraclass Correlation Coefficient*

| **Thought**  **Dimension** | **Idiographic Group**  **ICC [95%CI]** | **Nomothetic Group**  **ICC [95%CI]** |
| --- | --- | --- |
| Internal-External | 0.63 [0.33, 0.77] | 0.47 [0.34, 0.61] |
| Off-task | 0.36 [0.18, 0.56] | 0.19 [0.13, 0.28] |
| Freely moving | 0.44 [0.21, 0.70] | 0.29 [0.15, 0.40] |
| Goal-oriented | 0.58 [0.23, 0.82] | 0.45 [0.33, 0.58] |
| Sticky | 0.37 [0.15, 0.53] | 0.35 [0.22, 0.48] |
| Self-oriented | 0.54 [0.27, 0.73] | 0.36 [0.25, 0.51] |
| Others-oriented | 0.55 [0.27, 0.74] | 0.35 [0.26, 0.47] |
| Visual modality | 0.44 [0.27, 0.63] | 0.46 [0.36, 0.56] |
| Auditory modality | 0.36 [0.22, 0.54] | 0.48 [0.31, 0.64] |

**Supplementary** **Table S7**

*Main Effect of Task on Thought Dimension Ratings in each Participant and across Participants in the Idiographic Group Accounting for Number of Tasks*

| **Thought**  **Dimension** | **P1** | **P2** | **P3** | **P4** | **P5** | **P6** | **P7** | **Group** |
| --- | --- | --- | --- | --- | --- | --- | --- | --- |
| Internal-External | 34.81  *** | 81.95  *** | 10.74  * | 11.27  * | 47.18  *** | 1.50 | 2.58 | 75.68  *** |
| Task-relatedness | 44.66  *** | 29.49  *** | 21.06  *** | 17.04  ** | 98.87  *** | 2.09 | 8.21 | 82.40  *** |
| Freely moving | 4.48 | 27.11  *** | 31.71  *** | 16.50  ** | 28.31  *** | 4.17 | 0.96 | 53.72  *** |
| Goal-oriented | 22.43  *** | 11.55  * | 19.70  *** | 7.55 | 64.66  *** | 1.58 | 27.57  *** | 57.63  *** |
| Sticky | 8.92  * | 20.60  *** | 8.52 | 9.35 | 6.31 | 4.37 | 5.08 | 8.49 |
| Self-oriented | 1.89 | 1.91 | 9.86  * | 2.39 | 49.72  *** | 7.95 | 18.24  ** | 18.52  ** |
| Others-oriented | 5.64 | 38.93  *** | 27.32  *** | 9.25 | 73.42  *** | 75.02  *** | 6.99 | 27.09  *** |
| Visual modality | 56.81  *** | 45.77  *** | 29.24  *** | 15.42  * | 23.24  *** | 7.01 | 20.01  *** | 140.68  *** |
| Auditory modality | 86.44  *** | 43.79  *** | 39.02  *** | 9.75 | 2.39 | 3.07 | 9.54  * | 98.29  *** |

*Note: This reports the* $\chi$*^2^ values and their corresponding significance (*** significant after Bonferroni correction for multiple comparisons across nine thought dimensions and seven participants for the individual level analyses at p < .0008, ** significant after Bonferroni correction for multiple comparisons across nine thought dimensions at p < .006, * significant without correction for multiple comparison at p < .05) based on likelihood ratio tests using linear mixed effects analyses examining the main effect of task in predicting a thought dimension for each participant (P1 to P7) and across participants (at the group level) in the idiographic group (as shown above in separate columns).*

**Supplementary Table S8**

*Pairwise Contrasts as Follow-up to Significant Task Effect on Internally Oriented Thought in the Individual and Group Levels in the Idiographic Group*

|  | **beta** | **SE** | **95CI** | ***t*** | ***p* value** |
| --- | --- | --- | --- | --- | --- |
| **Group** |  |  |  |  |  |
| read vs video | -1.07 | 0.17 | [-1.56 -0.58] | -6.41 | < .001 |
| read vs write | 0.32 | 0.14 | [-0.09 0.72] | 2.30 | .030 |
| read vs web | -0.34 | 0.14 | [-0.75 0.08] | -2.37 | .027 |
| read vs games | 0.54 | 0.16 | [0.07 1.02] | 3.35 | .002 |
| read vs others | -0.04 | 0.15 | [-0.48 0.40] | -0.29 | .774 |
| video vs write | 1.39 | 0.20 | [0.80 1.97] | 6.98 | < .001 |
| video vs web | 0.74 | 0.20 | [0.15 1.31] | 3.73 | <.001 |
| video vs games | 1.61 | 0.21 | [0.99 2.23] | 7.67 | <.001 |
| video vs others | 1.02 | 0.21 | [0.40 1.65] | 4.80 | < .001 |
| write vs web | -0.65 | 0.17 | [-1.15 -0.16] | -3.88 | < .001 |
| write vs games | 0.23 | 0.19 | [-0.33 0.78] | 1.20 | .245 |
| write vs others | -0.36 | 0.17 | [-0.86 0.14] | -2.11 | .044 |
| web vs games | 0.88 | 0.18 | [0.36 1.40] | 5.00 | <.001 |
| web vs others | 0.29 | 0.18 | [-0.24 0.83] | 1.61 | .124 |
| games vs others | -0.59 | 0.20 | [-1.16 -0.01] | -3.00 | .005 |
| **Participant 1** |  |  |  |  |  |
| read vs video | -1.42 | 0.39 | [-2.47 -0.37] | -3.63 | <.001 |
| read vs write | 0.95 | 0.35 | [0.02 1.87] | 2.72 | .008 |
| read vs web | -2.00 | 0.54 | [-3.42 -0.58] | -3.74 | <.001 |
| video vs write | 2.37 | 0.48 | [1.10 3.64] | 4.99 | <.001 |
| video vs web | -0.58 | 0.58 | [-2.13 0.97] | -1.00 | .321 |
| write vs web | -2.95 | 0.59 | [-4.51 -1.39] | -5.02 | <.001 |
| **Participant 2** |  |  |  |  |  |
| read vs video | -3.07 | 0.32 | [-3.94 -2.20] | -9.62 | <.001 |
| read vs write | 0.70 | 0.41 | [-0.39 1.79] | 1.70 | .135 |
| read vs web | 0.09 | 0.34 | [-0.81 0.99] | 0.27 | .790 |
| video vs write | 3.77 | 0.45 | [2.58 4.96] | 8.47 | <.001 |
| video vs web | 3.16 | 0.46 | [1.94 4.39] | 6.94 | <.001 |
| write vs web | -0.61 | 0.53 | [-2.01 0.80] | -1.15 | .300 |
| **Participant 3** |  |  |  |  |  |
| read vs write | -0.20 | 0.36 | [-1.23 0.83] | -0.55 | .720 |
| read vs web | -0.03 | 0.37 | [-1.07 1.02] | -0.08 | .940 |
| read vs games | 0.44 | 0.31 | [-0.45 1.33] | 1.41 | .269 |
| read vs others | -0.73 | 0.43 | [-1.94 0.48] | -1.70 | .269 |
| write vs web | 0.17 | 0.38 | [-0.89 1.24] | 0.46 | .720 |
| write vs games | 0.64 | 0.34 | [-0.31 1.59] | 1.91 | .269 |
| write vs others | -0.53 | 0.42 | [-1.72 0.66] | -1.26 | .299 |
| web vs games | 0.47 | 0.33 | [-0.47 1.40] | 1.42 | .269 |
| web vs others | -0.70 | 0.46 | [-2.00 0.60] | -1.53 | .269 |
| games vs others | -1.17 | 0.39 | [-2.26 -0.07] | -3.03 | .028 |
| **Participant 5** |  |  |  |  |  |
| read vs video | -0.11 | 0.27 | [-0.95 0.73] | -0.42 | .782 |
| read vs write | 0.01 | 0.35 | [-1.02 1.05] | 0.04 | .968 |
| read vs web | 0.33 | 0.29 | [-0.56 1.22] | 1.12 | .528 |
| read vs games | 1.25 | 0.22 | [0.58 1.91] | 5.70 | <.001 |
| read vs others | -0.83 | 1.09 | [-4.06 2.39] | -0.77 | .621 |
| video vs write | 0.13 | 0.41 | [-1.17 1.43] | 0.31 | .813 |
| video vs web | 0.44 | 0.33 | [-0.59 1.47] | 1.35 | .462 |
| video vs games | 1.36 | 0.25 | [0.53 2.19] | 5.37 | <.001 |
| video vs others | -0.72 | 1.10 | [-3.97 2.53] | -0.66 | .640 |
| write vs web | 0.31 | 0.42 | [-0.97 1.60] | 0.75 | .621 |
| write vs games | 1.23 | 0.38 | [0.05 2.41] | 3.26 | .009 |
| write vs others | -0.85 | 1.13 | [-4.20 2.50] | -0.75 | .621 |
| web vs games | 0.92 | 0.26 | [0.14 1.70] | 3.53 | .003 |
| web vs others | -1.16 | 1.08 | [-4.36 2.03] | -1.08 | .528 |
| games vs others | -2.08 | 1.08 | [-5.28 1.12] | -1.93 | .166 |

*Note: This table reports the descriptive (beta estimate, standard error (SE) of the estimate) and statistics values (95% confidence interval (CI), t-value and p-value; *** p < .001, ** p < .01, * p < .05) of pairwise contrasts across all tasks at the group level and individual level for the idiographic group. We used the false discovery rate to correct for multiple comparison for each set of analysis. Pairwise comparisons were only implemented (and therefore reported in the table) if the group level analyses or specific participants showed a significant main effect of task. This summary applies to Supplementary Tables S7-15, for each of the nine thought dimensions.*

**Supplementary Table S9**

*Pairwise Contrasts as Follow-up to Significant Task Effect on Off-Task Thought in the Individual and Group Levels in the Idiographic Group*

|  | **beta** | **SE** | **95CI** | ***t*** | ***p* value** |
| --- | --- | --- | --- | --- | --- |
| **Group** |  |  |  |  |  |
| read vs video | 0.38 | 0.16 | [-0.09 0.84] | 2.39 | .026 |
| read vs write | 1.07 | 0.14 | [0.67 1.47] | 7.87 | <.001 |
| read vs web | 0.23 | 0.14 | [-0.18 0.64] | 1.65 | .125 |
| read vs games | -0.31 | 0.16 | [-0.77 0.16] | -1.93 | .073 |
| read vs others | 0.51 | 0.15 | [0.08 0.94] | 3.49 | .001 |
| video vs write | 0.69 | 0.19 | [0.14 1.25] | 3.66 | <.001 |
| video vs web | -0.14 | 0.19 | [-0.70 0.41] | -0.76 | .477 |
| video vs games | -0.68 | 0.20 | [-1.27 -0.09] | -3.39 | .002 |
| video vs others | 0.14 | 0.20 | [-0.46 0.74] | 0.67 | .501 |
| write vs web | -0.84 | 0.17 | [-1.33 -0.35] | -5.04 | <.001 |
| write vs games | -1.38 | 0.18 | [-1.91 -0.84] | -7.50 | <.001 |
| write vs others | -0.56 | 0.17 | [-1.05 -0.06] | -3.33 | .002 |
| web vs games | -0.54 | 0.17 | [-1.05 -0.03] | -3.11 | .003 |
| web vs others | 0.28 | 0.18 | [-0.24 0.81] | 1.57 | .134 |
| games vs others | 0.82 | 0.19 | [0.26 1.38] | 4.29 | <.001 |
| **Participant 1** |  |  |  |  |  |
| read vs video | 0.50 | 0.39 | [-0.54 1.53] | 1.28 | .304 |
| read vs write | 2.39 | 0.34 | [1.48 3.31] | 6.98 | <.001 |
| read vs web | 0.55 | 0.53 | [-0.85 1.95] | 1.05 | .357 |
| video vs write | 1.90 | 0.47 | [0.65 3.15] | 4.06 | <.001 |
| video vs web | 0.06 | 0.57 | [-1.47 1.58] | 0.10 | .923 |
| write vs web | -1.84 | 0.58 | [-3.38 -0.31] | -3.19 | .003 |
| **Participant 2** |  |  |  |  |  |
| read vs video | 0.39 | 0.09 | [0.09 0.69] | 4.21 | .003 |
| read vs write | 0.41 | 0.19 | [-0.11 0.92] | 2.17 | .041 |
| read vs web | -0.57 | 0.17 | [-1.01 -0.13] | -3.46 | .001 |
| video vs write | 0.02 | 0.18 | [-0.49 0.54] | 0.12 | .909 |
| video vs web | -0.96 | 0.17 | [-1.42 -0.50] | -5.70 | <.001 |
| write vs web | -0.98 | 0.24 | [-1.64 -0.32] | -4.08 | <.001 |
| **Participant 3** |  |  |  |  |  |
| read vs write | 1.40 | 0.37 | [0.36 2.44] | 3.82 | .001 |
| read vs web | 0.91 | 0.37 | [-0.15 1.96] | 2.44 | .038 |
| read vs games | 1.10 | 0.31 | [0.21 1.98] | 3.51 | .002 |
| read vs others | 1.58 | 0.42 | [0.38 2.78] | 3.76 | .001 |
| write vs web | -0.49 | 0.38 | [-1.56 0.57] | -1.31 | .284 |
| write vs games | -0.30 | 0.33 | [-1.25 0.64] | -0.92 | .452 |
| write vs others | 0.18 | 0.42 | [-1.00 1.36] | 0.44 | .664 |
| web vs games | 0.19 | 0.33 | [-0.74 1.12] | 0.58 | .628 |
| web vs others | 0.67 | 0.45 | [-0.61 1.96] | 1.50 | .274 |
| games vs others | 0.48 | 0.37 | [-0.59 1.56] | 1.29 | .284 |
| **Participant 4** |  |  |  |  |  |
| read vs video | -0.41 | 0.44 | [-1.72 0.91] | -0.92 | .494 |
| read vs write | 0.85 | 0.25 | [0.12 1.58] | 3.43 | .011 |
| read vs web | 0.43 | 0.26 | [-0.35 1.20] | 1.64 | .226 |
| read vs games | 0.58 | 0.63 | [-1.30 2.45] | 0.91 | .494 |
| read vs others | 0.31 | 0.22 | [-0.34 0.96] | 1.41 | .300 |
| video vs write | 1.25 | 0.44 | [-0.05 2.55] | 2.86 | .034 |
| video vs web | 0.83 | 0.41 | [-0.39 2.05] | 2.02 | .166 |
| video vs games | 0.98 | 0.74 | [-1.22 3.18] | 1.32 | .311 |
| video vs others | 0.71 | 0.44 | [-0.59 2.01] | 1.63 | .226 |
| write vs web | -0.42 | 0.25 | [-1.16 0.31] | -1.70 | .226 |
| write vs games | -0.27 | 0.64 | [-2.17 1.62] | -0.43 | .718 |
| write vs others | -0.54 | 0.22 | [-1.18 0.10] | -2.52 | .062 |
| web vs games | 0.15 | 0.64 | [-1.76 2.06] | 0.23 | .817 |
| web vs others | -0.12 | 0.24 | [-0.85 0.61] | -0.49 | .718 |
| games vs others | -0.27 | 0.62 | [-2.12 1.58] | -0.43 | .718 |
| **Participant 5** |  |  |  |  |  |
| read vs video | -0.28 | 0.28 | [-1.11 0.56] | -1.01 | .477 |
| read vs write | -0.31 | 0.34 | [-1.33 0.71] | -0.90 | .501 |
| read vs web | -1.17 | 0.29 | [-2.04 -0.29] | -3.98 | <.001 |
| read vs games | -2.09 | 0.22 | [-2.75 -1.44] | -9.54 | <.001 |
| read vs others | 0.15 | 1.06 | [-2.98 3.28] | 0.14 | .939 |
| video vs write | -0.03 | 0.42 | [-1.32 1.26] | -0.08 | .939 |
| video vs web | -0.89 | 0.34 | [-1.92 0.14] | -2.65 | .026 |
| video vs games | -1.82 | 0.26 | [-2.64 -1.00] | -6.90 | <.001 |
| video vs others | 0.43 | 1.07 | [-2.74 3.60] | 0.40 | .795 |
| write vs web | -0.86 | 0.42 | [-2.13 0.41] | -2.04 | .084 |
| write vs games | -1.79 | 0.38 | [-2.95 -0.62] | -4.64 | <.001 |
| write vs others | 0.46 | 1.10 | [-2.81 3.72] | 0.42 | .795 |
| web vs games | -0.93 | 0.26 | [-1.69 -0.17] | -3.63 | .001 |
| web vs others | 1.32 | 1.04 | [-1.77 4.41] | 1.27 | .345 |
| games vs others | 2.25 | 1.05 | [-0.86 5.35] | 2.15 | .071 |

*Refer to Note under Supplementary Table S8.*

**Supplementary Table S10**

*Pairwise Contrasts as Follow-up to Significant Task Effect on Freely Moving Thought in the Individual and Group Levels in the Idiographic Group*

|  | **beta** | **SE** | **95CI** | ***t*** | ***p* value** |
| --- | --- | --- | --- | --- | --- |
| **Group** |  |  |  |  |  |
| read vs video | 0.47 | 0.15 | [0.03 0.91] | 3.15 | .004 |
| read vs write | 0.34 | 0.13 | [-0.04 0.72] | 2.61 | .014 |
| read vs web | -0.22 | 0.14 | [-0.61 0.18] | -1.60 | .136 |
| read vs games | -0.67 | 0.15 | [-1.12 -0.22] | -4.41 | <.001 |
| read vs others | 0.29 | 0.14 | [-0.13 0.70] | 2.04 | .057 |
| video vs write | -0.13 | 0.18 | [-0.66 0.40] | -0.71 | .514 |
| video vs web | -0.69 | 0.18 | [-1.21 -0.16] | -3.81 | <.001 |
| video vs games | -1.14 | 0.19 | [-1.70 -0.58] | -5.95 | <.001 |
| video vs others | -0.18 | 0.19 | [-0.75 0.39] | -0.93 | .408 |
| write vs web | -0.56 | 0.16 | [-1.03 -0.09] | -3.49 | .001 |
| write vs games | -1.01 | 0.18 | [-1.53 -0.49] | -5.75 | <.001 |
| write vs others | -0.05 | 0.16 | [-0.52 0.42] | -0.33 | .743 |
| web vs games | -0.46 | 0.17 | [-0.94 0.03] | -2.74 | .010 |
| web vs others | 0.50 | 0.17 | [-0.00 1.01] | 2.94 | .006 |
| games vs others | 0.96 | 0.18 | [0.42 1.50] | 5.24 | <.001 |
| **Participant 2** |  |  |  |  |  |
| read vs video | 0.39 | 0.23 | [-0.26 1.04] | 1.71 | .146 |
| read vs write | 0.39 | 0.32 | [-0.46 1.24] | 1.24 | .261 |
| read vs web | -1.23 | 0.26 | [-1.93 -0.53] | -4.66 | <.001 |
| video vs write | 0.001 | 0.34 | [-0.91 0.91] | 0.00 | .997 |
| video vs web | -1.62 | 0.34 | [-2.54 -0.70] | -4.79 | <.001 |
| write vs web | -1.62 | 0.41 | [-2.71 -0.53] | -3.96 | <.001 |
| **Participant 3** |  |  |  |  |  |
| read vs write | 0.66 | 0.34 | [-0.30 1.61] | 1.95 | .087 |
| read vs web | 0.18 | 0.34 | [-0.79 1.14] | 0.53 | .599 |
| read vs games | -0.15 | 0.29 | [-0.96 0.66] | -0.54 | .599 |
| read vs others | 1.49 | 0.38 | [0.40 2.58] | 3.90 | <.001 |
| write vs web | -0.48 | 0.35 | [-1.45 0.50] | -1.38 | .241 |
| write vs games | -0.81 | 0.30 | [-1.67 0.05] | -2.68 | .021 |
| write vs others | 0.84 | 0.38 | [-0.24 1.91] | 2.21 | .058 |
| web vs games | -0.33 | 0.30 | [-1.18 0.52] | -1.11 | .336 |
| web vs others | 1.31 | 0.41 | [0.14 2.48] | 3.22 | .006 |
| games vs others | 1.64 | 0.34 | [0.67 2.62] | 4.87 | <.001 |
| **Participant 4** |  |  |  |  |  |
| read vs video | 1.07 | 0.38 | [-0.04 2.19] | 2.85 | .014 |
| read vs write | -0.01 | 0.21 | [-0.63 0.62] | -0.04 | .967 |
| read vs web | -0.25 | 0.22 | [-0.91 0.41] | -1.12 | .397 |
| read vs games | -0.76 | 0.54 | [-2.36 0.83] | -1.42 | .355 |
| read vs others | -0.16 | 0.19 | [-0.71 0.40] | -0.83 | .490 |
| video vs write | -1.08 | 0.37 | [-2.19 0.02] | -2.90 | .014 |
| video vs web | -1.32 | 0.35 | [-2.36 -0.28] | -3.78 | .003 |
| video vs games | -1.84 | 0.63 | [-3.71 0.03] | -2.92 | .014 |
| video vs others | -1.23 | 0.37 | [-2.33 -0.12] | -3.29 | .009 |
| write vs web | -0.24 | 0.21 | [-0.86 0.39] | -1.14 | .397 |
| write vs games | -0.76 | 0.54 | [-2.37 0.86] | -1.39 | 355 |
| write vs others | -0.15 | 0.18 | [-0.69 0.40] | -0.80 | .490 |
| web vs games | -0.52 | 0.55 | [-2.14 1.14] | -0.95 | .471 |
| web vs others | 0.09 | 0.21 | [-0.52 0.71] | 0.45 | .701 |
| games vs others | 0.61 | 0.53 | [-0.96 2.18] | 1.15 | .397 |
| **Participant 5** |  |  |  |  |  |
| read vs video | 0.59 | 0.33 | [-0.39 1.56] | 1.78 | .203 |
| read vs write | -0.34 | 0.39 | [-1.51 0.83] | -0.87 | .601 |
| read vs web | -0.58 | 0.34 | [-1.60 0.43] | -1.71 | .203 |
| read vs games | -1.08 | 0.26 | [-1.84 -0.31] | -4.18 | <.001 |
| read vs others | -0.44 | 1.20 | [-3.40 3.11] | -0.37 | .825 |
| video vs write | -0.93 | 0.50 | [-2.43 0.57] | -1.85 | .203 |
| video vs web | -1.17 | 0.41 | [-2.38 0.04] | -2.89 | .022 |
| video vs games | -1.66 | 0.32 | [-2.62 -0.71] | -5.19 | <.001 |
| video vs others | -1.02 | 1.22 | [-4.63 2.58] | -0.84 | .601 |
| write vs web | -0.24 | 0.50 | [-1.71 1.23] | -0.48 | .787 |
| write vs games | -0.73 | 0.46 | [-2.09 0.63] | -1.61 | .207 |
| write vs others | -0.09 | 1.25 | [-3.81 3.62] | -0.08 | .940 |
| web vs games | -0.49 | 0.29 | [-1.36 0.38] | -1.68 | .203 |
| web vs others | 0.15 | 1.18 | [-3.34 3.63] | 0.12 | .940 |
| games vs others | 0.64 | 1.18 | [-2.87 4.15] | 0.54 | .787 |

*Refer to Note under Supplementary Table S8.*

**Supplementary Table S11**

*Pairwise Contrasts as Follow-up to Significant Task Effect on Goal-Oriented* *Thought in the Individual and Group Levels in the Idiographic Group*

|  | **beta** | **SE** | **95CI** | ***t*** | ***p* value** |
| --- | --- | --- | --- | --- | --- |
| **Group** |  |  |  |  |  |
| read vs video | 0.33 | 0.17 | [-0.17 0.83] | 1.93 | .081 |
| read vs write | -0.73 | 0.14 | [-1.15 -0.31] | -5.11 | <.001 |
| read vs web | -0.20 | 0.15 | [-0.64 0.23] | -1.37 | .215 |
| read vs games | 0.14 | 0.17 | [-0.36 0.63] | 0.80 | .453 |
| read vs others | -0.86 | 0.16 | [-1.32 -0.40] | -5.51 | <.001 |
| video vs write | -1.06 | 0.20 | [-1.66 -0.46] | -5.21 | <.001 |
| video vs web | -0.53 | 0.20 | [-1.13 0.06] | -2.63 | .015 |
| video vs games | -0.19 | 0.22 | [-0.83 0.44] | -0.90 | .427 |
| video vs others | -1.19 | 0.22 | [-1.83 -0.54] | -5.40 | <.001 |
| write vs web | 0.53 | 0.18 | [0.02 1.05] | 3.03 | .005 |
| write vs games | 0.87 | 0.20 | [0.30 1.44] | 4.45 | <.001 |
| write vs others | -0.13 | 0.18 | [-0.65 0.40] | -0.70 | .481 |
| web vs games | 0.34 | 0.18 | [-0.20 0.87] | 1.84 | .089 |
| web vs others | -0.66 | 0.19 | [-1.21 -0.10] | -3.47 | .001 |
| games vs others | -0.99 | 0.20 | [-1.59 -0.40] | -4.89 | <.001 |
| **Participant 1** |  |  |  |  |  |
| read vs video | 0.69 | 0.43 | [-0.47 1.86] | 1.61 | .167 |
| read vs write | -1.62 | 0.39 | [-2.65 -0.59] | -4.17 | <.001 |
| read vs web | 0.01 | 0.60 | [-1.58 1.60] | 0.02 | .983 |
| video vs write | -2.31 | 0.52 | [-3.72 -0.90] | -4.41 | <.001 |
| video vs web | -0.68 | 0.65 | [-2.41 1.05] | -1.05 | .356 |
| write vs web | 1.63 | 0.66 | [-0.12 3.38] | 2.49 | .027 |
| **Participant 2** |  |  |  |  |  |
| read vs video | 0.56 | 0.23 | [-0.06 1.17] | 2.42 | .033 |
| read vs write | 0.81 | 0.27 | [0.08 1.53] | 2.96 | .020 |
| read vs web | -0.08 | 0.22 | [-0.67 0.52] | -0.34 | .736 |
| video vs write | -0.25 | 0.30 | [-0.56 1.06] | 0.83 | .490 |
| video vs web | -0.63 | 0.32 | [-1.48 0.22] | -1.98 | .074 |
| write vs web | -0.88 | 0.35 | [-1.82 0.05] | -2.51 | .033 |
| **Participant 3** |  |  |  |  |  |
| read vs write | -0.97 | 0.35 | [-1.96 0.01] | -2.79 | .019 |
| read vs web | -0.71 | 0.35 | [-1.71 0.29] | -2.01 | .073 |
| read vs games | -0.88 | 0.29 | [-1.70 -0.06] | -3.05 | .014 |
| read vs others | -1.71 | 0.37 | [-2.80 -0.62] | -4.57 | <.001 |
| write vs web | 0.26 | 0.36 | [-0.74 1.27] | 0.74 | .577 |
| write vs games | 0.09 | 0.30 | [-0.77 0.95] | 0.32 | .751 |
| write vs others | -0.74 | 0.37 | [-1.82 0.34] | -1.98 | .073 |
| web vs games | -0.17 | 0.30 | [-1.03 0.69] | -0.56 | .642 |
| web vs others | -1.00 | 0.39 | [-2.16 0.16] | -2.57 | 0.028 |
| games vs others | -0.83 | 0.32 | [-1.79 0.13] | -2.64 | .028 |
| **Participant 5** |  |  |  |  |  |
| read vs video | -0.04 | 0.22 | [-0.70 0.62] | -0.19 | .853 |
| read vs write | 0.19 | 0.27 | [-0.62 1.01] | 0.71 | .653 |
| read vs web | 0.47 | 0.23 | [-0.23 1.17] | 2.02 | .137 |
| read vs games | 1.20 | 0.17 | [0.68 1.72] | 6.96 | <.001 |
| read vs others | -0.20 | 0.85 | [-2.72 2.31] | -0.24 | .853 |
| video vs write | 0.23 | 0.33 | [-0.79 1.26] | 0.72 | .653 |
| video vs web | 0.51 | 0.26 | [-0.30 1.32] | 1.96 | .143 |
| video vs games | 1.24 | 0.20 | [0.59 1.89] | 6.13 | <.001 |
| video vs others | -0.16 | 0.86 | [-2.71 2.38] | -0.19 | .853 |
| write vs web | 0.28 | 0.33 | [-0.73 1.28] | 0.83 | .653 |
| write vs games | 1.01 | 0.30 | [0.08 1.93] | 3.36 | .006 |
| write vs others | -0.40 | 0.88 | [-3.02 2.22] | -0.45 | .815 |
| web vs games | 0.73 | 0.20 | [0.12 1.34] | 3.58 | .003 |
| web vs others | -0.67 | 0.84 | [-3.16 1.82] | -0.80 | .653 |
| game vs others | -1.40 | 0.84 | [-3.90 1.09] | -1.67 | .207 |
| **Participant 7** |  |  |  |  |  |
| read vs write | -2.15 | 0.65 | [-3.98 -0.31] | -3.33 | .005 |
| read vs web | -3.21 | 1.74 | [-8.13 1.72] | -1.85 | .110 |
| read vs games | 2.45 | 1.73 | [-2.45 7.35] | 1.42 | .197 |
| read vs others | -1.21 | 0.32 | [-2.16 -0.25] | -3.76 | .005 |
| write vs web | -1.06 | 1.83 | [-6.23 4.12] | -0.58 | .563 |
| write vs games | 4.60 | 1.77 | [-0.43 9.63] | 2.59 | .034 |
| write vs others | 0.94 | 0.64 | [-0.90 2.78] | 1.47 | .197 |
| web vs games | 5.66 | 2.43 | [-1.23 12.55] | 2.33 | .052 |
| web vs others | 2.00 | 1.71 | [-2.86 6.86] | 1.17 | .271 |
| game vs others | -3.66 | 1.73 | [-8.56 1.24] | -2.12 | .071 |

*Refer to Note under Supplementary Table S8.*

**Supplementary Table S12**

*Pairwise Contrasts as Follow-up to Significant Task Effect on Sticky* *Thought in the Individual and Group Levels in the Idiographic Group*

|  | **beta** | **SE** | **95CI** | ***t*** | ***p* value** |
| --- | --- | --- | --- | --- | --- |
| **Participant 1** |  |  |  |  |  |
| read vs video | -0.53 | 0.37 | [-1.54 0.49] | -1.41 | .327 |
| read vs write | -1.01 | 0.34 | [-1.91 -0.11] | -3.00 | .018 |
| read vs web | -0.06 | 0.52 | [-1.45 1.32] | -0.12 | .901 |
| video vs write | -0.49 | 0.46 | [-1.71 0.74] | -1.07 | .431 |
| video vs web | 0.46 | 0.57 | [-1.04 1.96] | 0.82 | .498 |
| write vs web | 0.95 | 0.57 | [-0.57 2.47] | 1.66 | .295 |
| **Participant 2** |  |  |  |  |  |
| read vs video | 0.60 | 0.16 | [0.16 1.05] | 3.77 | .002 |
| read vs write | 0.49 | 0.22 | [-0.08 1.07] | 2.27 | .036 |
| read vs web | -0.30 | 0.18 | [-0.77 0.18] | -1.67 | .117 |
| video vs write | -0.11 | 0.23 | [-0.73 0.51] | -0.47 | .641 |
| video vs web | -0.90 | 0.22 | [-1.53 -0.27] | -3.86 | .001 |
| write vs web | -0.79 | 0.28 | [-1.53 -0.05] | -2.84 | .010 |

*Refer to Note under Supplementary Table S8.*

**Supplementary Table S13**

*Pairwise Contrasts as Follow-up to Significant Task Effect on Self-Oriented* *Thought in the Individual and Group Levels in the Idiographic Group*

|  | **beta** | **SE** | **95CI** | ***t*** | ***p* value** |
| --- | --- | --- | --- | --- | --- |
| **Group** |  |  |  |  |  |
| read vs video | -0.07 | 0.16 | [-0.54 0.39] | -0.46 | .693 |
| read vs write | -0.17 | 0.13 | [-0.56 0.21] | -1.33 | .276 |
| read vs web | -0.10 | 0.14 | [-0.50 0.30] | -0.75 | .620 |
| read vs games | -0.45 | 0.15 | [-0.90 0.00] | -2.92 | .022 |
| read vs others | 0.29 | 0.14 | [-0.13 0.71] | 2.03 | .107 |
| video vs write | -0.10 | 0.19 | [-0.66]0.45 | -0.54 | .639 |
| video vs web | -0.03 | 0.19 | [-0.58 0.52] | -0.15 | .880 |
| video vs games | -0.38 | 0.20 | [-0.96 0.21] | -1.89 | .126 |
| video vs others | 0.36 | 0.20 | [-0.23 0.96] | 1.79 | .139 |
| write vs web | 0.07 | 0.16 | [-0.40 0.54] | 0.46 | .693 |
| write vs games | -0.28 | 0.18 | [-0.80 0.25] | -1.54 | .206 |
| write vs others | 0.46 | 0.16 | [-0.01 0.94] | 2.86 | .022 |
| web vs games | -0.35 | 0.17 | [-0.84 0.14] | -2.09 | .107 |
| web vs others | 0.39 | 0.17 | [-0.12 0.90] | 2.26 | .091 |
| games vs others | 0.74 | 0.19 | [0.19 1.29] | 3.97 | .001 |
| **Participant 3** |  |  |  |  |  |
| read vs write | -0.01 | 0.34 | [-0.99 0.96] | -0.04 | .969 |
| read vs web | -0.42 | 0.35 | [-1.40 0.57] | -1.20 | .283 |
| read vs games | 0.36 | 0.30 | [-0.48 1.20] | 1.22 | .283 |
| read vs others | 0.80 | 0.41 | [-0.35 1.96] | 1.98 | .123 |
| write vs web | -0.40 | 0.35 | [-1.41 0.60] | -1.14 | .283 |
| write vs games | 0.37 | 0.32 | [-0.53 1.28] | 1.17 | .283 |
| write vs others | 0.82 | 0.40 | [-0.31 1.95] | 2.05 | .123 |
| web vs games | 0.78 | 0.31 | [-0.10 1.66] | 2.50 | .066 |
| web vs others | 1.22 | 0.44 | [-0.02 2.47] | 2.79 | .058 |
| games vs others | 0.44 | 0.37 | [-0.60 1.49] | 1.20 | .283 |
| **Participant 5** |  |  |  |  |  |
| read vs video | -0.64 | 0.35 | [-1.67 0.39] | -1.85 | .137 |
| read vs write | 0.31 | 0.41 | [-0.91 1.54] | 0.76 | .557 |
| read vs web | -1.29 | 0.36 | [-2.35 -0.23] | -3.61 | .001 |
| read vs games | -1.88 | 0.27 | [-2.69 -1.08] | -6.96 | <.001 |
| read vs others | -0.70 | 1.25 | [-4.40 2.99] | -0.56 | .663 |
| video vs write | 0.96 | 0.53 | [-0.62 2.53] | 1.80 | .137 |
| video vs web | -0.65 | 0.43 | [-1.93 0.62] | -1.52 | .216 |
| video vs games | -1.24 | 0.34 | [-2.25 -0.23] | -3.65 | .001 |
| video vs others | -0.06 | 1.27 | [-3.82 3.70] | -0.05 | .962 |
| write vs web | -1.61 | 0.52 | [-3.15 -0.06] | -3.08 | .007 |
| write vs games | -2.20 | 0.48 | [-3.62 -0.77] | -4.57 | <.001 |
| write vs others | -1.02 | 1.30 | [-4.89 2.85] | -0.78 | .557 |
| web vs games | -0.59 | 1.31 | [-1.50 0.32] | -1.93 | .137 |
| web vs others | 0.59 | 1.22 | [-3.03 4.21] | 0.49 | .673 |
| game vs others | 1.18 | 1.23 | [-2.47 4.83] | 0.96 | .506 |
| **Participant 7** |  |  |  |  |  |
| read vs write | -1.15 | 0.41 | [-2.32 0.03] | -2.78 | .030 |
| read vs web | 0.88 | 1.11 | [-2.26 4.02] | 0.79 | .612 |
| read vs games | 1.15 | 1.10 | [-1.97 4.27] | 1.05 | .494 |
| read vs others | 0.48 | 0.21 | [-0.14 1.09] | 2.25 | .093 |
| write vs web | 2.03 | 1.16 | [-1.27 5.32] | 1.74 | .166 |
| write vs games | 2.30 | 1.13 | [-0.90 5.50] | 2.04 | .107 |
| write vs others | 1.63 | 0.42 | [0.44 2.81] | 3.91 | .001 |
| web vs games | 0.27 | 1.55 | [-4.11 4.66] | 0.18 | .860 |
| web vs others | -0.40 | 1.09 | [-3.49 2.69] | -0.37 | .792 |
| game vs others | -0.68 | 1.10 | [-3.80 2.45] | -0.61 | .676 |

*Refer to Note under Supplementary Table S8.*

**Supplementary Table S14**

*Pairwise Contrasts as Follow-up to Significant Task Effect on Other-Oriented* *Thought in the Individual and Group Levels in the Idiographic Group*

|  | **beta** | **SE** | **95CI** | ***t*** | ***p* value** |
| --- | --- | --- | --- | --- | --- |
| **Group** |  |  |  |  |  |
| read vs video | -0.48 | 0.19 | [-1.03 0.07] | -2.57 | .022 |
| read vs write | -0.55 | 0.15 | [-1.00 -0.10] | -3.60 | .004 |
| read vs web | -0.43 | 0.16 | [-0.90 0.02] | -2.75 | .018 |
| read vs games | -0.52 | 0.18 | [-1.05 0.01] | -2.86 | .017 |
| read vs others | 0.11 | 0.17 | [-0.39 0.60] | 0.63 | .796 |
| video vs write | -0.07 | 0.22 | [-0.73 0.58] | -0.32 | .879 |
| video vs web | 0.05 | 0.22 | [-0.60 0.69] | 0.22 | .879 |
| video vs games | -0.04 | 0.24 | [-0.73 0.66] | -0.15 | .879 |
| video vs others | 0.59 | 0.24 | [-0.12 1.29] | 2.45 | .027 |
| write vs web | 0.12 | 0.19 | [-0.43 0.67] | 0.63 | .796 |
| write vs games | 0.04 | 0.21 | [-0.58 0.65] | 0.17 | .879 |
| write vs others | 0.66 | 0.19 | [0.10 1.22] | 3.46 | .004 |
| web vs games | -0.08 | 0.20 | [-0.66 0.49] | -0.43 | .879 |
| web vs others | 0.54 | 0.20 | [-0.06 1.13] | 2.66 | .020 |
| games vs others | 0.62 | 0.22 | [-0.02 1.26] | 2.85 | .017 |
| **Participant 2** |  |  |  |  |  |
| read vs video | 0.07 | 0.21 | [-0.50 0.64] | 0.32 | 0.75 |
| read vs write | -1.50 | 0.25 | [-2.18 -0.83] | -5.91 | <.001 |
| read vs web | 0.36 | 0.21 | [-0.19 0.91] | 1.75 | .121 |
| video vs write | -1.57 | 0.28 | [-2.32 -0.82] | -5.56 | <.001 |
| video vs web | 0.29 | 0.30 | [-0.50 1.09] | 0.99 | .387 |
| write vs web | 1.86 | 0.33 | [0.99 2.74] | 5.69 | <.001 |
| **Participant 3** |  |  |  |  |  |
| read vs write | -0.90 | 0.50 | [-2.32 0.51] | -1.81 | .103 |
| read vs web | -1.28 | 0.51 | [-2.72 0.15] | -2.53 | .030 |
| read vs games | -0.36 | 0.42 | [-1.57 0.84] | -0.86 | .436 |
| read vs others | 1.16 | 0.57 | [-0.46 2.78] | 2.04 | .072 |
| write vs web | -0.38 | 0.51 | [-1.83 1.07] | -0.74 | .457 |
| write vs games | 0.54 | 0.45 | [-0.74 1.82] | 1.21 | .288 |
| write vs others | 2.06 | 0.56 | [0.46 3.66] | 3.67 | .002 |
| web vs games | 0.92 | 0.44 | [-0.34 2.18] | 2.07 | .072 |
| web vs others | 2.44 | 0.60 | [0.70 4.18] | 4.05 | .001 |
| games vs others | 1.52 | 0.50 | [0.07 2.97] | 3.05 | .011 |
| **Participant 5** |  |  |  |  |  |
| read vs video | -0.73 | 0.36 | [-1.81 0.35] | -2.01 | .078 |
| read vs write | -2.99 | 0.44 | [-4.28 -1.70] | -6.89 | <.001 |
| read vs web | -1.63 | 0.38 | [-2.75 -0.51] | -4.33 | <.001 |
| read vs games | -1.72 | 0.29 | [-2.57 -0.88] | -6.04 | <.001 |
| read vs others | -2.54 | 1.32 | [-6.44 1.37] | -1.93 | .083 |
| video vs write | -2.26 | 0.56 | [-3.92 -0.60] | -4.05 | .001 |
| video vs web | -0.90 | 0.45 | [-2.24 0.44] | -2.00 | .078 |
| video vs games | -0.99 | 0.36 | [-2.05 0.07] | -2.77 | .018 |
| video vs others | -1.81 | 1.34 | [-5.78 2.16] | -1.35 | .243 |
| write vs web | 1.36 | 0.55 | [-0.27 2.99] | 2.48 | .030 |
| write vs games | 1.27 | 0.51 | [-0.23 2.77] | 2.52 | .030 |
| write vs others | 0.46 | 1.38 | [-3.63 4.54] | 0.33 | .783 |
| web vs games | -0.09 | 0.32 | [-1.05 0.87] | -0.28 | .783 |
| web vs others | -0.90 | 1.29 | [-4.73 2.92] | 0.70 | .605 |
| game vs others | -0.82 | 1.30 | [-4.67 3.04] | -0.63 | .612 |
| **Participant 6** |  |  |  |  |  |
| read vs video | -3.07 | 0.37 | [-4.14 -1.99] | -8.21 | <.001 |
| read vs web | 0.28 | 0.26 | [-0.46 1.02] | 1.09 | .558 |
| read vs games | 0.20 | 0.94 | [-2.45 2.86] | 0.22 | .947 |
| read vs others | 0.32 | 0.40 | [-0.81 1.44] | 0.80 | .704 |
| video vs web | 3.35 | 0.42 | [2.17 4.53] | 8.10 | <.001 |
| video vs games | 3.27 | 1.01 | [0.42 6.12] | 3.25 | .003 |
| video vs others | 3.38 | 0.54 | [1.86 4.91] | 6.29 | <.001 |
| web vs games | -0.08 | 0.96 | [-2.80 2.64] | -0.08 | .937 |
| web vs others | 0.04 | 0.45 | [-1.24 1.31] | 0.08 | .937 |
| games vs others | 0.11 | 1.00 | [-2.72 2.94] | 0.11 | .937 |

*Refer to Note under Supplementary Table S8.*

**Supplementary Table S15**

*Pairwise Contrasts as Follow-up to Significant Task Effect on Visual Thought in the Individual and Group Levels in the Idiographic Group*

|  | **beta** | **SE** | **95CI** | ***t*** | ***p* value** |
| --- | --- | --- | --- | --- | --- |
| **Group** |  |  |  |  |  |
| read vs video | -1.24 | 0.17 | [-1.75 -0.73] | -7.13 | <.001 |
| read vs write | 0.84 | 0.14 | [0.42 1.26] | 5.87 | <.001 |
| read vs web | -0.76 | 0.15 | [-1.19 -0.33] | -5.17 | <.001 |
| read vs games | -0.24 | 0.17 | [-0.74 0.25] | -1.44 | 0.16 |
| read vs others | -0.39 | 0.16 | [-0.85 0.07] | -2.48 | .018 |
| video vs write | 2.08 | 0.21 | [1.47 2.69] | 10.07 | <.001 |
| video vs web | 0.48 | 0.20 | [-0.12 1.08] | 2.34 | .024 |
| video vs games | 1.00 | 0.22 | [0.35 1.64] | 4.56 | <.001 |
| video vs others | 0.85 | 0.22 | [0.20 1.51] | 3.83 | <.001 |
| write vs web | -1.60 | 0.18 | [-2.12 -1.09] | -9.17 | <.001 |
| write vs games | -1.08 | 0.20 | [-1.66 -0.51] | -5.55 | <.001 |
| write vs others | -1.23 | 0.18 | [-1.75 -0.71] | -6.93 | <.001 |
| web vs games | 0.52 | 0.18 | [-0.02 1.06] | 2.85 | .007 |
| web vs others | 0.37 | 0.19 | [-0.18 0.93] | 1.98 | .055 |
| games vs others | -0.15 | 0.20 | [-0.74 0.45] | -0.71 | .475 |
| **Participant 1** |  |  |  |  |  |
| read vs video | -2.50 | 0.43 | [-3.66 -1.34] | -5.76 | <.001 |
| read vs write | 1.56 | 0.38 | [0.55 2.57] | 4.09 | <.001 |
| read vs web | -0.57 | 0.58 | [-2.13 0.99] | -0.98 | .331 |
| video vs write | 4.06 | 0.53 | [2.66 5.46] | 7.73 | <.001 |
| video vs web | 1.93 | 0.64 | [0.23 3.62] | 3.03 | .003 |
| write vs web | -2.13 | 0.64 | [-3.84 -0.42] | -3.32 | .002 |
| **Participant 2** |  |  |  |  |  |
| read vs video | -2.13 | 0.39 | [-3.18 -1.08] | -5.41 | <.001 |
| read vs write | 0.65 | 0.47 | [-0.60 1.91 | 1.38 | .168 |
| read vs web | -1.30 | 0.39 | [-2.33 -0.28] | -3.37 | .002 |
| video vs write | 2.78 | 0.52 | [1.39 4.18] | 5.32 | <.001 |
| video vs web | 0.83 | 0.55 | [-0.63 2.29] | 1.51 | .159 |
| write vs web | -1.95 | 0.61 | [-3.58 -0.33] | -3.21 | .002 |
| **Participant 3** |  |  |  |  |  |
| read vs write | 0.80 | 0.43 | [-0.41 2.00] | 1.88 | .077 |
| read vs web | -1.29 | 0.44 | [-2.51 -0.07] | -2.99 | .008 |
| read vs games | 0.02 | 0.37 | [-1.01 1.06] | 0.06 | .953 |
| read vs others | -0.94 | 0.50 | [-2.35 0.48] | -1.88 | .077 |
| write vs web | -2.09 | 0.44 | [-3.32 -0.85] | -4.77 | <.001 |
| write vs games | -0.78 | 0.39 | [-1.89 0.33] | -1.98 | .077 |
| write vs others | -1.74 | 0.49 | [-3.13 -0.35] | -3.55 | .002 |
| web vs games | 1.31 | 0.38 | [0.22 2.40] | 3.41 | .003 |
| web vs others | 0.35 | 0.54 | [-1.17 1.87] | 0.65 | .575 |
| games vs others | -0.96 | 0.45 | [-2.24 0.32] | -2.14 | .068 |
| **Participant 4** |  |  |  |  |  |
| read vs video | -0.55 | 0.51 | [-2.06 0.95] | -1.09 | .377 |
| read vs write | 0.26 | 0.28 | [-0.58 1.10] | 0.92 | .449 |
| read vs web | -0.70 | 0.30 | [-1.59 0.19] | -2.34 | .075 |
| read vs games | 1.19 | 0.72 | [-0.96 3.33] | 1.64 | .220 |
| read vs others | 0.07 | 0.25 | [-0.68 0.81] | 0.27 | .789 |
| video vs write | 0.81 | 0.50 | [-0.68 2.31] | 1.62 | .220 |
| video vs web | -0.15 | 0.47 | [-1.55 1.25] | -0.31 | .789 |
| video vs games | 1.71 | 0.85 | [-0.78 4.26] | 2.05 | .125 |
| video vs others | 0.62 | 0.50 | [-0.87 2.11] | 1.23 | .328 |
| write vs web | -0.96 | 0.28 | [-1.80 -0.12] | -3.38 | .013 |
| write vs games | 0.93 | 0.73 | [-1.25 3.10] | 1.27 | .328 |
| write vs others | -0.19 | 0.25 | [-0.93 0.54] | -0.79 | .500 |
| web vs games | 1.89 | 0.74 | [-0.30 4.07] | 2.57 | .055 |
| web vs others | 0.77 | 0.28 | [-0.06 1.60] | 2.74 | .050 |
| games vs others | -1.12 | 0.71 | [-3.23 1.00] | -1.57 | .220 |
| **Participant 5** |  |  |  |  |  |
| read vs video | -0.26 | 0.20 | [-0.84 0.32] | -1.33 | .400 |
| read vs write | 0.22 | 0.24 | [-0.48 0.92] | 0.92 | .536 |
| read vs web | -0.49 | 0.20 | [-1.10 0.12] | -2.39 | .069 |
| read vs games | -0.68 | 0.15 | [-1.14 -0.22] | -4.42 | <.001 |
| read vs others | -0.41 | 0.72 | [-2.53 1.72] | -0.57 | .714 |
| video vs write | 0.48 | 0.30 | [-0.42 1.37] | 1.59 | .285 |
| video vs web | -0.23 | 0.24 | [-0.95 0.49] | -0.94 | .536 |
| video vs games | -0.42 | 0.19 | [-0.99 0.15] | -2.20 | .088 |
| video vs others | -0.15 | 0.73 | [-2.31 2.01] | -0.20 | .900 |
| write vs web | -0.70 | 0.30 | [-1.58 0.18] | -2.38 | .069 |
| write vs games | -0.90 | 0.27 | [-1.71 -0.09] | -3.30 | .009 |
| write vs others | -0.62 | 0.75 | [-2.85 1.60] | -0.83 | .554 |
| web vs games | -0.19 | 0.18 | [-0.71 0.33] | -1.10 | .513 |
| web vs others | 0.08 | 0.70 | [-2.01 2.17] | 0.11 | .909 |
| game vs others | 0.27 | 0.71 | [-1.83 2.37] | 0.39 | .808 |
| **Participant 7** |  |  |  |  |  |
| read vs write | 0.95 | 0.51 | [-0.49 2.39] | 1.87 | .130 |
| read vs web | -1.68 | 1.35 | [-5.51 2.15] | -1.25 | .306 |
| read vs games | -2.35 | 1.34 | [-6.14 1.45] | -1.76 | .134 |
| read vs others | -0.92 | 0.28 | [-1.71 -0.13] | -3.31 | .005 |
| write vs web | -2.63 | 1.42 | [-6.66 1.39] | -1.85 | .130 |
| write vs games | -3.30 | 1.37 | [-7.18 0.58] | -2.41 | .056 |
| write vs others | -1.87 | 0.52 | [-3.35 -0.40] | -3.60 | .004 |
| web vs games | -0.67 | 1.88 | [-6.01 4.67] | -0.35 | .724 |
| web vs others | 0.76 | 1.32 | [-2.99 4.51] | 0.57 | .629 |
| game vs others | 1.43 | 1.34 | [-2.38 5.23] | 1.06 | .362 |

*Refer to Note under Supplementary Table S8.*

**Supplementary Table S16**

*Pairwise Contrasts as Follow-up to Significant Task Effect on Auditory* *Thought in the Individual and Group Levels in the Idiographic Group*

|  | **beta** | **SE** | **95CI** | ***t*** | ***p* value** |
| --- | --- | --- | --- | --- | --- |
| **Group** |  |  |  |  |  |
| read vs video | 0.95 | 0.15 | [0.52 1.39] | 6.47 | <.001 |
| read vs write | -0.40 | 0.12 | [-0.77 -0.04] | -3.24 | .002 |
| read vs web | 0.53 | 0.13 | [0.16 0.91] | 4.18 | <.001 |
| read vs games | -0.19 | 0.15 | [-0.61 0.24] | -1.29 | .218 |
| read vs others | 0.38 | 0.14 | [-0.02 0.77] | 2.79 | .007 |
| video vs write | -1.36 | 0.18 | [-1.87 -0.84] | -7.70 | <.001 |
| video vs web | -0.42 | 0.18 | [-0.93 0.09] | -2.41 | .020 |
| video vs games | -1.14 | 0.19 | [-1.69 -0.59] | -6.11 | <.001 |
| video vs others | -0.58 | 0.19 | [-1.14 -0.02] | -3.05 | .004 |
| write vs web | 0.94 | 0.15 | [0.49 1.38] | 6.18 | <.001 |
| write vs games | 0.21 | 0.17 | [-0.28 0.71] | 1.27 | .218 |
| write vs others | 0.78 | 0.13 | [0.33 1.23] | 5.08 | <.001 |
| web vs games | -0.72 | 0.16 | [-1.19 -0.26] | -4.57 | <.001 |
| web vs others | -0.16 | 0.16 | [-0.64 0.32] | -0.97 | .332 |
| games vs others | 0.56 | 0.18 | [0.05 1.08] | 3.22 | .002 |
| **Participant 1** |  |  |  |  |  |
| read vs video | 2.43 | 0.32 | [1.57 3.29] | 7.52 | <.001 |
| read vs write | -0.84 | 0.28 | [-1.59 -0.09] | -2.99 | .004 |
| read vs web | 2.75 | 0.43 | [1.61 0.39] | 6.41 | <.001 |
| video vs write | -3.27 | 0.39 | [-4.31 -2.23] | -8.39 | <.001 |
| video vs web | 0.32 | 0.47 | [-0.92 1.57] | 0.69 | .492 |
| write vs web | 3.59 | 0.47 | [2.34 4.85] | 7.61 | <.001 |
| **Participant 2** |  |  |  |  |  |
| read vs video | 1.87 | 0.31 | [1.04 2.70] | 6.01 | <.001 |
| read vs write | -0.69 | 0.37 | [-1.69 0.30] | -1.85 | .089 |
| read vs web | 0.48 | 0.31 | [-0.33 1.29] | 1.58 | .115 |
| video vs write | -2.57 | 0.41 | [-3.67 -1.46] | -6.20 | <.001 |
| video vs web | -1.39 | 0.44 | [-2.55 -0.24] | -3.20 | .003 |
| write vs web | 1.17 | 0.48 | [-0.11 2.25] | 2.44 | .023 |
| **Participant 3** |  |  |  |  |  |
| read vs write | 0.09 | 0.32 | [-0.83 1.01] | 0.28 | .868 |
| read vs web | 0.09 | 0.33 | [-0.84 1.02] | 0.28 | .868 |
| read vs games | -0.45 | 0.28 | [-1.23 0.34] | -1.60 | .158 |
| read vs others | 1.76 | 0.38 | [0.68 2.83] | 1.64 | <.001 |
| write vs web | 0.00 | 0.33 | [-0.95 0.95] | 0.00 | .999 |
| write vs games | -0.54 | 0.30 | [-1.38 0.31] | -1.81 | .121 |
| write vs others | 1.66 | 0.37 | [0.61 2.72] | 4.47 | <.001 |
| web vs games | -0.54 | 0.29 | [-1.37 0.29] | -1.84 | .121 |
| web vs others | 1.66 | 0.41 | [0.51 2.82] | 4.10 | <.001 |
| games vs others | 2.20 | 0.34 | [1.23 3.17] | 6.59 | <.001 |

*Refer to Note under Supplementary Table S8.*

**Supplementary Table S17**

*Main Effect of Task on Thought Dimension Ratings and Follow-up Pairwise Contrasts in the Nomothetic Group*

| **Pairwise Contrasts** | Int-Ext  $\chi$^2^ = 19.04 ** | Task  $\chi$^2^ = 31.36 ** | Free $\chi$^2^ = 21.27 ** | Goal  $\chi$^2^ = 94.15 ** | Sticky  $\chi$^2^ = 9.15 | Self  $\chi$^2^ = 21.42 ** | Others  $\chi$^2^ = 18.88 ** | Visual  $\chi$^2^ = 66.57 ** | Aud  $\chi$^2^ = 36.11 ** |
| --- | --- | --- | --- | --- | --- | --- | --- | --- | --- |
| **Re-Vi** |  |  |  | pos  *** |  |  |  | neg  *** |  |
| **Re-Wr** |  |  |  |  |  |  |  | pos  *** | neg  ** |
| **Re-We** |  |  |  |  |  |  |  | neg  * | pos  ** |
| **Re-Ga** |  | pos  *** |  | neg  *** |  |  |  |  |  |
| **Re-Ot** |  | pos  *** |  | neg  *** |  | neg  ** |  |  |  |
| **Vi-Wr** |  |  |  | neg  *** |  |  |  | pos  *** |  |
| **Vi-We** |  |  | neg  * | neg  *** |  |  |  | pos  * | pos  ** |
| **Vi-Ga** |  | pos  *** |  | neg  *** |  |  |  | pos  * |  |
| **Vi-Ot** |  | pos  ** |  | neg  *** |  |  | pos  * | pos  *** |  |
| **Wr-We** | neg  * |  |  |  |  |  | neg  * | neg  *** | pos  *** |
| **Wr-Ga** | neg  * | pos  *** |  | neg  * |  |  |  | neg  ** | pos  ** |
| **Wr-Ot** |  | pos  ** |  | neg  ** |  |  |  | neg  ** | pos  * |
| **We-Ga** |  | pos  *** | pos  ** | neg  ** |  |  |  |  |  |
| **We-Ot** |  | pos  ** | pos  ** | neg  *** |  |  | pos  * | pos  * | neg  ** |
| **Ga-Ot** |  |  |  |  |  | neg  ** |  |  |  |

*Note: The first column reports the* $\chi$*^2^ values and their corresponding significance (** significant after correction for multiple comparison at p < .006, * significant without correction for multiple comparison at p < .05) based on likelihood ratio tests using linear mixed effects analyses examining the main effect of task in predicting a thought dimension across participants in the nomothetic group. For all other columns, this table reports pairwise contrasts for a given pair of tasks and their corresponding FDR corrected significance level (*** p < .001, ** p < .01, * p < .05) separately for each thought dimension. Each column shows each pairwise comparison across the five task categories, with the first task in each pair in the column heading as the reference. Positive effects indicates that the level of thought dimension was significantly higher in first task in each pair compared to the second task. For example, there were significantly higher levels of goal oriented thoughts during reading compared to video watching. Re = reading/studying; Vi = watching videos; Wr = writing; We = web surfing; Ga = games; Ot = other cognitively demanding tasks.*

**Supplementary Table S18**

*Comparison of Classification Performance of each participant for Task Predicting Ongoing Thoughts Within Participants in the Idiographic Group*

| **Performance Metrics** | Internal-External | Task-related-ness | Freely moving | Goal-oriented | Sticky | Self-oriented | Others-oriented | Visual modality | Auditory modality |
| --- | --- | --- | --- | --- | --- | --- | --- | --- | --- |
| P1 |  |  |  |  |  |  |  |  |  |
| MCC | 0.24 | 0.23 | 0.07 | 0.12 | 0.12 | 0.31 | 0.36 | 0.30 | 0.26 |
| BA | 0.62 | 0.61 | 0.54 | 0.56 | 0.56 | 0.65 | 0.68 | 0.65 | 0.63 |
| P2 |  |  |  |  |  |  |  |  |  |
| MCC | 0.27 | 0.75 | 0.07 | 0.75 | 0.62 | 0.39 | 0.43 | 0.05 | 0.23 |
| BA | 0.63 | 0.87 | 0.54 | 0.88 | 0.81 | 0.70 | 0.71 | 0.52 | 0.61 |
| P3 |  |  |  |  |  |  |  |  |  |
| MCC | 0.35 | 0.11 | 0.23 | 0.27 | 0.23 | 0.08 | 0.23 | 0.05 | 0.37 |
| BA | 0.68 | 0.56 | 0.62 | 0.64 | 0.62 | 0.54 | 0.62 | 0.53 | 0.69 |
| P4 |  |  |  |  |  |  |  |  |  |
| MCC | 0.40 | 0.49 | 0.22 | 0.29 | 0.18 | 0.11 | 0.05 | 0.16 | 0.41 |
| BA | 0.70 | 0.75 | 0.61 | 0.64 | 0.60 | 0.56 | 0.53 | 0.58 | 0.70 |
| P5 |  |  |  |  |  |  |  |  |  |
| MCC | 0.19 | 0.15 | 0.18 | 0.31 | 0.15 | 0.14 | 0.16 | 0.32 | 0.18 |
| BA | 0.60 | 0.57 | 0.59 | 0.65 | 0.58 | 0.57 | 0.58 | 0.66 | 0.59 |
| P6 |  |  |  |  |  |  |  |  |  |
| MCC | 0.52 | 0.59 | 0.61 | 0.50 | 0.11 | -0.04 | 0.61 | 0.54 | 0.44 |
| BA | 0.76 | 0.79 | 0.81 | 0.75 | 0.53 | 0.31 | 0.80 | 0.77 | 0.72 |
| P7 |  |  |  |  |  |  |  |  |  |
| MCC | 0.06 | 0.07 | 0.10 | 0.04 | 0.55 | 0.63 | 0.81 | 0.04 | 0.03 |
| BA | 0.53 | 0.53 | 0.55 | 0.52 | 0.77 | 0.82 | 0.90 | 0.52 | 0.52 |
| Group |  |  |  |  |  |  |  |  |  |
| MCC | 0.29 | 0.34 | 0.21 | 0.32 | 0.28 | 0.23 | 0.38 | 0.21 | 0.27 |
| BA | 0.64 | 0.67 | 0.61 | 0.66 | 0.64 | 0.59 | 0.69 | 0.60 | 0.64 |

*Note: MCC = Matthew’s Correlation Coefficient; BA = Balanced Accuracy. The classification performance as indexed by two performance metrics are reported for each participant (P1 to P7) and the overall group in the idiographic group, representing the detection of a given thought dimension based on task-at-hand. Each value represents the mean classification performance across 100 iterations.*

**Supplementary Table S19**

*Comparison of Classification Performance of Task Predicting Ongoing Thoughts Within and Across Participants in the Idiographic Group*

|  | **Within Participant** | | **Across Participant** | |
| --- | --- | --- | --- | --- |
| **Thought Dimension** | **MCC** | **BA** | **MCC** | **BA** |
| Internal-External | 0.29 | 0.64 | 0.00 | 0.50 |
| Task-relatedness | 0.34 | 0.67 | 0.33 | 0.67 |
| Freely moving | 0.21 | 0.61 | 0.02 | 0.51 |
| Goal-oriented | 0.32 | 0.66 | 0.07 | 0.54 |
| Sticky | 0.28 | 0.64 | 0.23 | 0.62 |
| Self-oriented | 0.23 | 0.59 | 0.26 | 0.63 |
| Others-oriented | 0.38 | 0.69 | 0.25 | 0.62 |
| Visual modality | 0.21 | 0.60 | 0.05 | 0.52 |
| Auditory modality | 0.27 | 0.64 | 0.02 | 0.51 |

*Note: MCC = Matthew’s Correlation Coefficient; BA = Balanced Accuracy. The classification performance as indexed by two performance metrics are reported for the idiographic group, representing the detection of a given thought dimension based on task-at-hand. Each value represents the mean (and standard deviation) classification performance across the 100 iterations. Classification performance for the within-participant models was significantly better than the across-participant models at p < .001 for all thought dimensions, except for self-oriented thought in which the opposite pattern was observed at p < .001. The values reported for the within participant models are identical to those reported in Table 3.*
